# Butterfly‐Shaped Guest Molecules Enable Tunable Room‐Temperature Phosphorescence in Host‐Guest Doped Systems

**DOI:** 10.1002/advs.202507940

**Published:** 2025-10-08

**Authors:** Zongyong Lou, Wenhui Feng, Kaining Wang, Kun Gong, Guangyu Wen, Denghui Ji

**Affiliations:** ^1^ Hebei Petroleum University of Technology Cheng De 067000 P. R. China; ^2^ School of Chemical Engineering and Technology Tianjin University Tianjin 300354 P. R. China; ^3^ College of Physics and Hebei Advanced Thin Film Laboratory Hebei Normal University Shijiazhuang 050024 P. R. China; ^4^ Science College Shijiazhuang University Shijiazhuang 050035 P. R. China

**Keywords:** host‐guest doped systems, multi‐color tunable afterglow materials, phosphorescent material

## Abstract

Organic room‐temperature phosphorescence (RTP) materials have attracted significant interest due to their potential in optoelectronics and anti‐counterfeiting. However, achieving multicolor‐tunable and long‐lived RTP with simple, low‐cost systems remains challenging. Herein, a facile host‐guest doping strategy is developed to realize efficient and color‐tunable RTP by embedding butterfly‐shaped triphenylamine‐based guest molecules (TPA, DBD, and DBDBD) into various host matrices (e.g., TPP, BPP, or CA). The doped crystals exhibit distinct afterglow colors (green to yellow) and prolonged long‐persistent luminescence (LPL) (from 1 to 6 s of afterglow time) and phosphorescence lifetimes up to 763.33 ms, governed by host‐guest energy transfer and intersystem crossing enhancement. Density functional theory (DFT) calculations reveal that the guest's electron‐donating ability and the host's heavy‐atom effect (e.g., P in TPP) synergistically promote charge separation and suppress non‐radiative decay. Notably, DBDBD:TPP shows the longest LPL (6 s of afterglow time) due to optimal energy level alignment and strong intermolecular interactions. By leveraging the time‐ and color‐dependent afterglow, applications in multilevel information encryption and anti‐counterfeiting are demonstrated, where encrypted messages are dynamically revealed under UV excitation. This work provides a simple yet versatile approach to designing low‐cost, multicolor RTP materials for advanced photonic applications.

## Graphical abstract

Butterfly‐shaped triphenylamine‐based guest molecules (DBDBD) doped into rigid host matrices (TPP, CA) achieve efficient, multicolor‐tunable room‐temperature phosphorescence (RTP). The host‐guest synergy enables ultralong afterglow (up to 4 s) and quantum yields (Φp = 3.42%), with applications in dynamic anti‐counterfeiting and information encryption. DFT and photophysical studies reveal enhanced intersystem crossing and suppressed non‐radiative decay via heavy‐atom effects and optimal energy alignment.

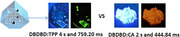

## 1 Introduction

Time‐dependent dynamic room‐temperature phosphorescent (RTP) materials have garnered significant attention in organic light‐emitting diodes (OLEDs), biological imaging, information encryption, and chemical sensing due to their low cost, good biocompatibility, and ease of structural modification.^[^ ^1^, ^2^, ^3^, ^4^, ^5^, ^6^, ^7^, ^8^, ^9^, ^10^ ^]^ However, the low spin–orbit coupling (SOC) constant, the oxygen quenching effect, and the solvent‐induced non‐radiative transitions in pure organic molecules make it challenging to achieve RTP,^[^ ^11^, ^12^ ^]^ especially in aqueous solutions. Enhancing singlet‐triplet intersystem crossing (ISC) and reducing non‐radiative transitions are crucial for achieving RTP.^[^ ^13^, ^14^, ^15^ ^]^ Traditional strategies,^[^ ^16^, ^17^, ^18^, ^19^, ^20^, ^21^, ^22^, ^23^, ^24^, ^25^ ^]^ such as introducing heavy atoms, heteroatoms, aromatic carbonyls, sulfones, and other structures^[^ ^17^ ^]^ to enhance the ISC process or constructing a rigid environment through complex crystal engineering,^[^ ^21^ ^]^ polymers,^[^ ^23^, ^24^ ^]^ or host‐guest doping^[^ ^18^ ^]^ systems to suppress non‐radiative transitions. However, challenges remain in achieving systems that combine simple synthesis, low cost, and high performance.

Aniline organic small molecules,^[^ ^26^, ^27^, ^28^ ^]^ Triphenylamine (TPA),^[^ ^26^ ^]^ Triphenylphosphine (TPP),^[^ ^29^ ^]^ and their derivatives^[^ ^30^, ^31^, ^32^ ^]^ are well known for their strong intramolecular electron transfer ability, large conjugated systems, good crystallization capability, thermal stability, rich optical properties, and strong hole transport capacity. These characteristics make them widely used in constructing hosted molecules with luminescent behavior. The TPP system has many ISC channels and possesses abundant intermolecular C─H…*π* interactions, effectively suppressing non‐radiative transitions. If the guest material has a similar molecular structure to the host material, the introduction of the guest will not significantly change the lattice structure of the host molecule. It is crucial to identify high‐performance, cost‐effective, and readily available guest molecules with well‐matched energy levels and excellent crystallizability.

**Figure 1:**
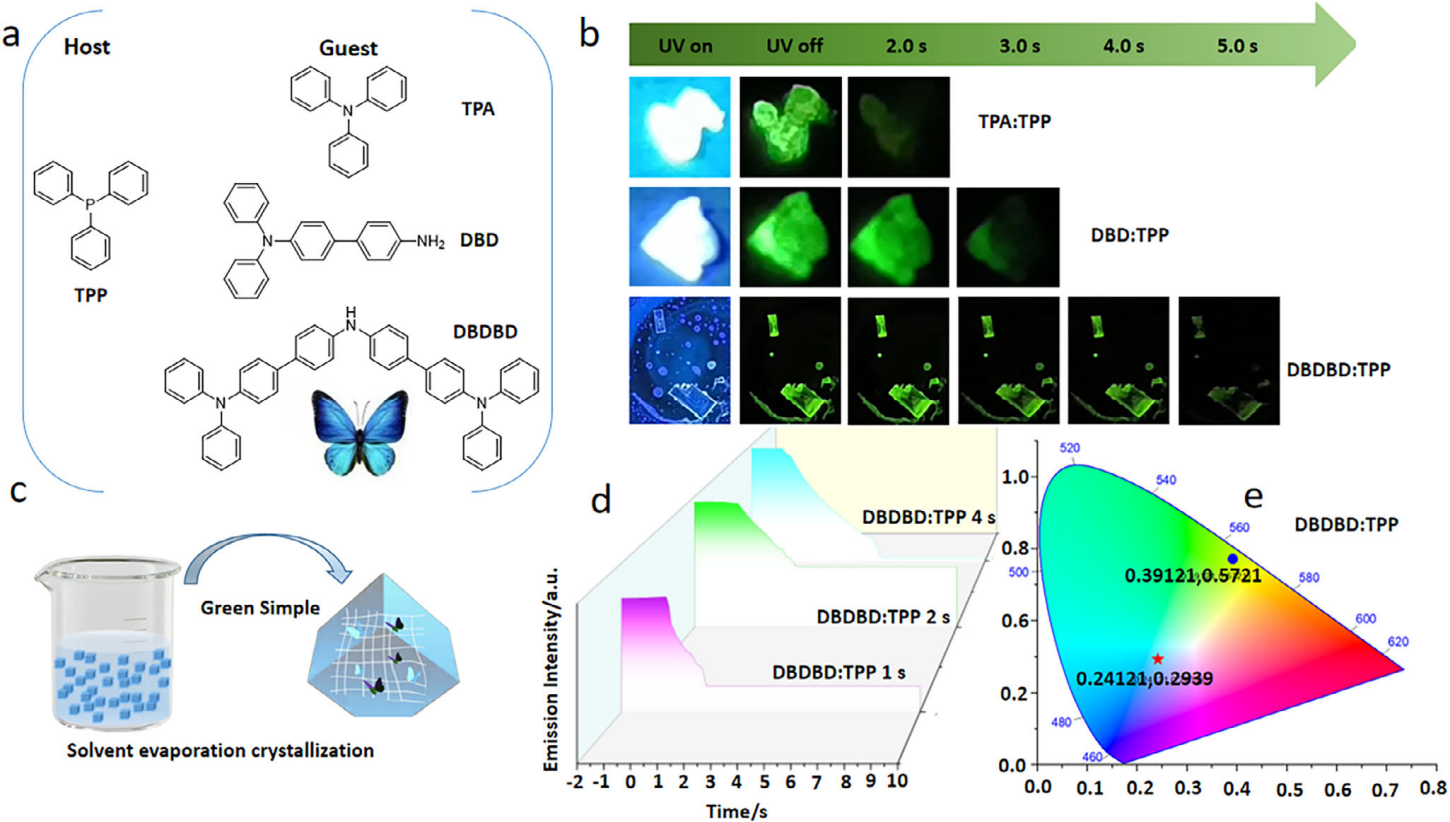
a) Chemical structures of TPA, DBD, DBDBD, and TPP along with b) the LPL photographs of the doped crystal (TPA: TPP, DBD: TPP, and DBDBD: TPP) under ambient conditions (excitation: 365 nm). c) Preparation of 1% guest‐doped host crystals via a “green and simple” solvent evaporation crystallization method. d) Semi‐logarithmic plot of the emission decay profiles for the two doped crystals, showing photoluminescence (PL) from −2 to 0 s and long‐persistent luminescence (LPL) from 0 to 10 s after excitation is turned off (excitation wavelength: 365 nm; excitation power: 10 mW; excitation time: 2 s; and sample temperature: 300 K). e) Emission colors in the CIE 1931 chromaticity diagram: PL (star) and LPL (triangle) for DBDBD: TPP.

We adopted a general host‐guest doping strategy, which could easily construct a multi‐color, adjustable, and efficient RTP system without complex molecular design. By simply doping the butterfly‐shaped guest N4‐(4′‐diphenylamino‐1,1′‐biphenyl‐4‐yl‐N4',N4'‐diphenyl‐[1,1′‐biphenyl]‐4,4′‐diamine (DBDBD) in various organic small molecule hosts (TPP, TPA, CA), we have used an extremely simple solvent evaporation crystallization method to achieve effective host‐guest doping, which successfully induced the long afterglow performance of the organic doped crystals under various environmental conditions. Through the energy transfer of host‐guest materials, the doped materials DBDBD:TPP and DBDBD:CA emitted two distinct colors of afterglow. As shown in **Figures** **1** and S1 (Supporting Information), after excitation with a 365 nm UV lamp, DBDBD:TPP exhibited a green afterglow lasting up to 4 s (Figure 1b), while DBDBD:CA exhibited a yellow afterglow lasting up to 2 s (Figure S1b, Supporting Information), as confirmed by their respective emission decay curves (Figure 1d for DBDBD:TPP and Figure S1c, Supporting Information for DBDBD:CA) and CIE diagrams (Figure 1e; Figure S1d, Supporting Information). With this strategy, we realized a simple and effective screening mechanism for developing different long‐persistent luminescence (LPL) materials. In contrast, our butterfly‐shaped DBDBD guest enables dynamic color switching (green to yellow) and prolonged LPL (up to 4 s of afterglow time) through simple host variations (e.g., TPP vs CA), bypassing the need for intricate supramolecular engineering or cross‐linked polymers. To highlight the application advantages of crystal materials with two different afterglow colors, we used a binary encoding method for information encryption and a printing method for anti‐counterfeiting applications.

## 2 Results and Discussion

### 2.1 Color‐Tunable RTP Achieved

We developed a multi‐color‐tunable, pure organic RTP system via the host‐guest doping strategy. As shown in Figure 1a, several butterfly‐shaped triphenylamine derivatives (TPA, DBD, and DBDBD) were selected as guests, while the readily crystallizable TPP served as the host material. All doped crystals (TPA:TPP, DBD:TPP, and DBDBD:TPP) exhibited visible afterglow upon UV removal. As shown in Figure 1b,d, the afterglow time duration lengthened with enhanced electron‐donating capability in the guest: TPA:TPP (1 s), DBD:TPP (2 s), and DBDBD:TPP (4 s). Structural and chemical characterization (e.g., nuclear magnetic resonance (^1^H NMR), Figure S2, Supporting Information; high‐resolution mass spectrometry (HRMS), Figure S3, Supporting Information; High‐Performance Liquid Chromatography (HPLC), Figure S4, Supporting Information) confirmed DBDBD's high purity (> 99%) and molecular consistency. Notably, DBDBD:TPP showed a green afterglow time 4 s, as corroborated by the emission decay and CIE diagrams in Figure 1d,e, indicating that the guest's electronic structure could effectively tune the afterglow properties.

### 2.2 Photophysical Properties and Inferred Energy Transfer Mechanism

**Figure 2:**
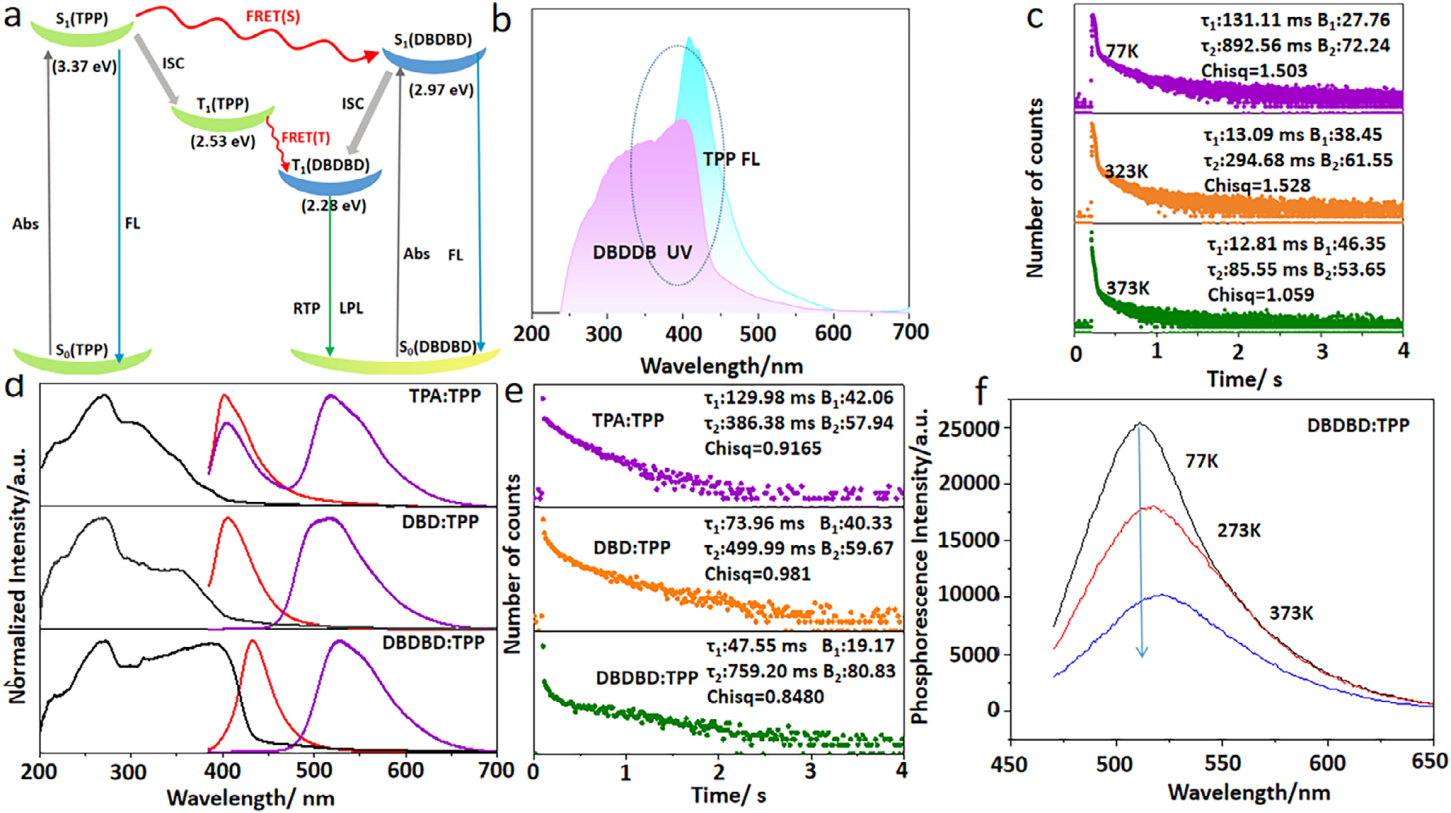
a) FRET processes of the doped⁃crystals DBDBD:TPP. b) Overlap between the UV–vis absorption spectrum of DBDBD (burgundy red) and the fluorescence emission spectrum of TPP (blue). The Dotted line area indicates the spectral overlap, suggesting possible energy transfer. c) Phosphorescence emission decay spectra of the DBDBD:TPP. (Normalized phosphorescence spectra recorded from 77 to 373 K under 365 nm excitation). d) UV–vis absorption (black) spectra, fluorescence (red), and phosphorescence (purple) emission spectra of the TPA:TPP, DBD:TPP and DBDBD:TPP powder (excitation at λ_max_ of absorption), all were measured at room temperature. e) Phosphorescence emission decay spectra for the doped crystals TPA:TPP, DBD:TPP and TPA:TPP, DBD:TPP and DBDBD:TPP (excitation: 365 nm). f) Temperature dependence of phosphorescence intensity for DBDBD:TPP. (Normalized phosphorescence spectra recorded from 77 to 373 K under 365 nm excitation).

The host‐guest crystal materials with afterglow properties were selected for further photophysical investigation, among which 1 mol% guest molecules were doped in TPP. We measured the UV–vis absorption spectroscopy, fluorescence spectroscopy, phosphorescence spectroscopy of the monomer molecule of TPA, DBD, DBDBD and the doped crystals of TPA:TPP, DBD:TPP, DBDBD:TPP, as shown in Figure S5 (Supporting Information) and **Figure** **2**d. Influenced by the compound short π connections, the absorption peaks of the monomer powder were situated at 293, 345, and 375 nm, respectively (Figure S5a–c, Supporting Information). The fluorescence emission spectra of TPA, DBD, and DBDBD were situated at 405, 410, and 460 nm, respectively. The phosphorescence emission spectra of TPA, DBD, and DBDBD were situated at 505, 517, and 546 nm, respectively (Figure S5d–f, Supporting Information). Under 77 K, guest TPA, DBD and DBDBD showed a strong green phosphorescence (500–550 nm) and a shorter phosphorescence life times (TPA:16.32 ms, DBD:19.95 ms and DBDBD: 36.19 ms) (Figure S6, Supporting Information). However, the maximum absorption wavelengths, fluorescence wavelengths and phosphorescence wavelengths of TPA and DBA were shorter than those of DBDBD (Figure S5, Supporting Information), mainly caused by the difference in the electron supply capacity of molecules, this preliminary observation strongly suggested that the guest's molecular energy levels were being effectively tuned.^[^ ^26^ ^]^

A central question is whether the observed ultralong afterglow originates solely from direct excitation of the guest, or also benefits significantly from host‐guest interactions. Although the DBDBD guest has strong absorption at 365 nm compared to the TPP host, which suggests a direct excitation pathway, the dramatically extended phosphorescence lifetime in the doped system (from 36.19 ms (Figure S6, Supporting Information) for pure DBDBD to 759.20 ms (Figure 2e) for DBDBD:TPP) strongly suggests that the host matrix plays a role beyond merely providing a rigid confinement.

Multiple lines of evidence collectively support the existence of effective host‐guest interactions that enhance triplet state population efficiency. We have performed detailed analysis of the singlet energy levels (Table S1, Supporting Information; Figure 2a), confirming that TPP's triplet energies (2.53 eV) is higher than DBDBD's (2.28 eV), satisfying the essential condition for Förster resonance energy transfer (FRET).^[^ ^9^, ^25^, ^32^, ^33^ ^]^ The intermolecular distance between host and guest molecules (<10 nm in doped crystalline systems) coupled with spectral overlap (TPP emission matching DBDBD absorption, as shown Figure 2b) fulfills the essential criteria for FRET.^[^ ^9^, ^25^, ^32^, ^33^ ^]^ The fluorescence decay curves (Figure S7, Supporting Information) show a single‐exponential decay (2.75 ns lifetime) at 420 nm for DBDBD:TPP, along with complete quenching of TPP fluorescence and enhanced DBDBD fluorescence emission. These observations provide direct evidence of efficient energy transfer from host to guest. Most notably, a dramatic phosphorescence lifetime increase from 36.19 ms (pure components DBDBD) to 759.20 ms (DBDBD:TPP doped system) – representing a 20 × enhancement that unequivocally demonstrates efficient host‐guest energy transfer and triplet state stabilization (Figure 1e). The phosphorescence decay curves of doped crystals (e.g., DBDBD:TPP) exhibit two components. A long‐lived component (759.20 ms, 80.83%) attributed to guest triplet states stabilized by host‐guest interactions. A short‐lived component (47.55 ms, 19.17%) linked to host‐mediated energy transfer. The dominance of the long‐lived component in DBDBD:TPP confirms efficient host‐to‐guest energy transfer. This consistent pattern across all guest molecules confirms that the excitation energy is primarily absorbed by the host matrix and then efficiently transferred to the guest, which subsequently emits via phosphorescence.

Therefore, we infer that the highly efficient intersystem crossing (ISC) and ultralong phosphorescence lifetime originate from a synergistic effect of host‐guest interactions: the rigid crystalline environment effectively suppresses non‐radiative decay, while potential interactions such as energy transfer between host and guest optimize the generation and stabilization of triplet excitons.^[^ ^25^, ^26^, ^32^, ^33^ ^]^

### 2.3 Guest‐Dependent RTP Performance and Trends

The UV–vis absorption spectroscopy, fluorescence spectroscopy, phosphorescence spectroscopy, lifetimes and phosphorescence quantum yields (Φp) exhibited a remarkable and systematic dependence on the guest molecule. As shown Figure 2d and Figure S5 (Supporting Information), when the guest changes from TPA to DBDBD, the ultraviolet absorption peak of the doped material gradually redshifts (Figure 2d black). Particularly when the guest molecule is DBDBD, the UV absorption peak exhibits a redshift from 330 nm (TPA) to 400 nm and the maximum fluorescence and phosphorescence wavelengths of TPA:TPP and DBD:TPP are significantly shorter than those of DBDBD:TPP (Figure 2d red and purple), which further demonstrates that modifying the guest structure significantly alters the photophysical properties of the material. The host matrix modules in doped‐crystals (TPP) have no absorption under 365 nm excitation (Figure S8, Supporting Information), while the TPA, DBD and DBDBD module has significant absorption and then emits the characteristic fluorescence and phosphorescence (Figure S7, Supporting Information). The host‐guest doped crystal materials produced a green phosphorescence with a wavelength ranging from 520 to 550 nm.

The data reveals a compelling trend: strengthening the guest's electron‐donating capability concurrently redshifts the phosphorescence, prolongs its lifetime, and enhances the quantum yield. Notably, the phosphorescence lifetime of the doped crystals shows a continuous increase, from 386.38 ms for TPA: TPP to 499.99 ms for DBD: TPP and further to 759.20 ms for DBDBD: TPP (Figure 2e), this is also consistent with the increasing trend of afterglow time (TPA:TPP 1 s, DBD:TPP 2 s, DBDBD:TPP 4 s, as shown Figure 1b). More importantly, the TPA:TPP, DBD:TPP and DBDBD:TPP systems exhibit Φp values of 1.24%, 2.55% and 3.42%, respectively (**Table** **1**; Figure S9, Supporting Information). This progressive enhancement mirrors the trend observed in phosphorescence lifetimes and afterglow durations, confirming that the increased electron‐donating capability of the guest molecules (TPA < DBD < DBDBD) prolongs the triplet state lifetime and improves the radiative transition probability.^[^ ^9^, ^26^ ^]^ The Φp of 3.42%, phosphorescence lifetime of 759.20 ms and the most redshifted emission at 546 nm achieved by DBDBD:TPP indicate a balance between efficient ISC (facilitated by host‐guest charge transfer) and suppressed non‐radiative decay (enabled by the rigid crystalline environment).^[^ ^9^, ^26^, ^34^, ^35^ ^]^

**Table 1:** Room‐Temperature Phosphorescence Performance, SOC, τ_p_, Afterglow time and Emission Color of TPA: TPP, DBD: TPP, DBDBD: TPP, DBDBD:BPP, DBDBD:CA and DBDBD:2MoBPA.

Guest	Host	SOC[cm^−1^]	τ_p_[ms]	Afterglow time[s]	Φ_p_[%]	Emission color
TPA	TPP	0.84 (S_1_–T_1_)	386.38	1	1.24	Green
DBD	TPP	1.05 (S_1_–T_1_)	499.99	2	2.55	Green
DBDBD	TPP	1.31 (S_1_–T_1_)	759.20	4	3.42	Green
DBDBD	BPP	3.30 (S_1_–T_1_)	763.33	6	3.58	Green
DBDBD	CA	0.15 (S_1_–T_1_)	444.84	2	–	Yellow
DBDBD	2MoBPA	0.58 (S_1_–T_1_)	165.66	<0.1	–	Green

**Figure 3:**
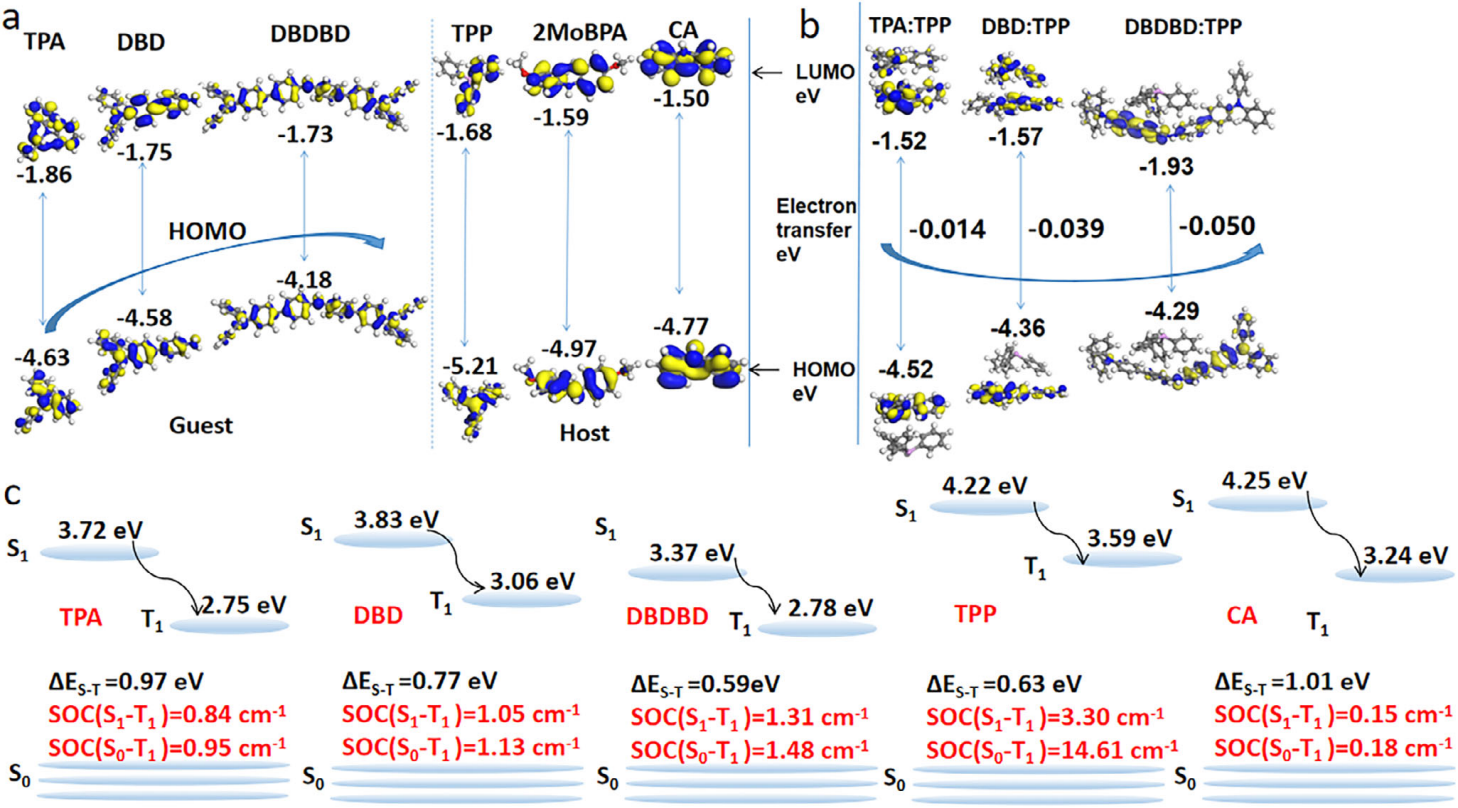
a) Calculation of ground and excited state geometry, HOMO‐LUMO energy level distribution of TPA, DBD, DBDBD, TPP, 2MoBPA and CA. b) Electron transfer between host and guest and HOMO‐LUMO of TPA:TPP, DBD:TPP and DBDBD:TPP using DMol3 program. (Calculation methods in Supporting Information Characterization data). c) Energy level diagram showing the singlet (S_1_) and triplet (T_1_) states with their energy gap (ΔE_S1‐T1_) and SOC constants.

We attribute this systematic enhancement to a synergistic mechanism where the guest's molecular design promotes efficient intersystem crossing (ISC), which is further facilitated by the host's heavy‐atom effect. Density functional theory (DFT) calculations provide crucial insights into this mechanism (**Figure** **3**, Table 1; Tables S3–S9, Supporting Information).

First, the redshift in emission is directly associated with a reduction in the guest's triplet energy gap (ΔE_S‐T_), a result of its raised HOMO energy level as donor strength increases.^[^ ^26^, ^36^ ^]^ As shown in Figure 3a, Table 1 and Table S2 (Supporting Information), the highest occupied molecular orbitals (HOMOs) and the lowest unoccupied molecular orbitals (LUMOs), energy level diagram showing the singlet (S_1_) and triplet (T_1_) states with their energy gap (ΔE_S–T_) and spin–orbit coupling (SOC) constants of TPA, DBD, DBDBD, TPP and CA by DFT. The calculation results indicate that the HOMO energy levels of TPA, DBD, and DBDBD rise (increase) as the donor strength increases (HOMO energy levels of TPA, DBD, DBDBD are −4.63, −4.58 and −4.18 eV, respectively. The energy gaps between singlet and triplet states (ΔE_S1–T1_) decrease with the increasing electron‐donating ability of the guest (TPA: 0.97 eV → DBDBD: 0.59 eV). This synergy enhances ISC and prolongs phosphorescence lifetimes.^[^ ^26^ ^]^

Second, promoted charge transfer (CT) character: Natural transition orbital (NTO) analysis reveals that stronger donors strengthen electron transfer from the guest to the host upon excitation (Figure 3b). The electron transfer between the host and guest was calculated, showing that DBDBD:TPP transferred more electrons (0.050 eV) than DBD: TPP (0.039 eV) and TPA:TPP (0.014 eV). This enhanced CT character promotes the formation of a triplet state, which is known to facilitate ISC.^[^ ^26^, ^36^ ^]^

Three, strong SOC (Figure 3c; Tables S2–S9, Supporting Information): The heavy atom effect from the brominated TPP host provides a powerful intrinsic driver for ISC. The calculated SOC values between singlet and triplet states increased dramatically with guest donor strength, from 0.84 cm^−1^ (TPA) to 1.31 cm^−1^ (DBDBD) (Table 1 and Figure 3c). This 1.5‐fold enhancement in SOC directly correlates with the observed elongation of the phosphorescence lifetime, as a higher SOC constant accelerates the ISC rate.

The biexponential phosphorescence decay profiles (Figure 2e) provide further kinetic evidence for this host‐guest synergy. The transient phosphorescence spectra reveal that the RTP emission at 520 nm exhibits a biexponential decay with lifetimes of TPA:TPP (386.38 ms, 57.94% and 129.98 ms, 42.06%), DBD:TPP (499.99 ms, 59.67% and 73.96 ms, 40.33%) and DBDBD:TPP (759.20 ms, 80.83% and 47.55 ms, 19.17%) indicating that this prominent RTP emission from the excited TPA:TPP, DBD:TPP and DBDBD:TPP crystal originates from both the triplet states of TPA, DBD and DBDBD and TPP. More interestingly, as the donor groups transition from TPA to DBD and then to DBDBD, the long‐lived component gradually increases (TPA:TPP < 57.94% DBD:TPP 59.67% < DBDBD:TPP 80.83%) while the short‐lived component decreases (TPA:TPP 42.06% > DBD:TPP > 40.33%> DBDBD:TPP 19.17%), which is one of the key reasons for the significant enhancement in afterglow duration.

This mechanism is further supported by variable‐temperature phosphorescence measurements. we examined the temperature‐dependent phosphorescence of TPA:TPP, DBDBD:BPP (Figure S10, Supporting Information) and DBDBD:TPP over the range of 77–373 K (Figure 2f). All systems exhibited a monotonic decrease in phosphorescence intensity with increasing temperature (Figure 2f; Figure S10, Supporting Information), The phosphorescence lifetime also decreased with rising temperature for all systems (Figure 2c). For example, as shown Figure 2c DBDBD:TPP showed a significant reduction in lifetime from 892.56 ms at 77 K to 759.20 ms at room temperature (300 K) and further reduced to a shorter lifetime 294.68 ms at 353 K and 85.55 ms at 373K. This temperature‐activated non‐radiative decay process is a hallmark of RTP systems and rules out a long‐persistent luminescence (LPL) mechanism based on charge trapping/detrapping.^[^ ^6^ ^]^ Notably, DBDBD:TPP retained the highest residual phosphorescence intensity at 373 K, highlighting the robustness of its host‐guest stabilization. These findings further validate our design strategy of employing rigid crystalline matrices to suppress vibrational quenching while promoting efficient intersystem crossing.^[^ ^15^, ^21^, ^26^ ^]^ This behavior supports the role of thermally activated vibrational modes in accelerating non‐radiative decay, a characteristic feature of RTP systems.^[^ ^6^, ^26^ ^]^ Temperature‐dependent measurements of the afterglow time for DBDBD:TPP revealed a clear decline with increasing temperature, further confirming the reduced stabilization of triplet states at elevated temperatures. This reduction is attributed to enhanced molecular motions and increased susceptibility to oxygen quenching.

### 2.4 Versatility and Superiority of the Strategy Validated

To further investigate the properties of the DBDBD molecule, we used DBDBD as the guest and selected other easily crystallizable host materials (TPA, 9H‐carbazole (CA) and Methoxybenzophenone (2MoBPA) were used as the host materials to prepare doped materials (DBDBD:TPA, DBDBD:2MoBPA and DBDBD:CA) (Figure S1, Supporting Information). Unfortunately, when TPA and 2MoBPA were used as hosts, the performance of DBDBD:TPA and DBDBD:2MoBPA were poor, with extremely short afterglow time (Figure S1b,c, Supporting Information). When CA was used as the host, an orange afterglow of DBDBD:CA was observed, but the afterglow time was only 2 s (Figure S1b,c, Supporting Information), primarily due to limitations in energy level matching between the materials. Based on the phosphorescence decay curves (Figure S11, Supporting Information), the phosphorescence lifetimes of the doped crystalline powders ranged from 55.57 ms (DBDBD:TPA), 165.66 ms (DBDBD:2MoBPA) to 444.84 ms (DBDBD:CA), resulting in the multi‐color afterglow (DBDBD:TPA and DBDBD:2MoBPA, green < 1 s, DBDBD:CA, yellow < 2 s) that lasted only 0.1–2 s under ambient conditions (Figure S1b, Supporting Information). In addition, the phosphorescence peaks of the TPA‐based materials obviously redshifted compared to those of the TPP‐based materials, likely due to the effect of phosphorus atoms.

To investigate the origin of the variation in afterglow colors, we calculated the energies of the singlet and triplet states and the SOC constants (Figure 3c, Table 1; Tables S2–S9, Supporting Information). In DBDBD:TPP, the smaller energy gap ΔE_S1‐T1_ (0.63 eV: TPP) promotes efficient intersystem crossing (ISC) to the triplet state (T_1_) of DBDBD, resulting in a green afterglow (520–550 nm). In DBDBD:CA, the larger energy gap (1.01 eV: CA) weaker shift the emission to yellow (550–600 nm) owing to relaxed triplet‐state geometry and reduced ISC efficiency.

Hosts with phosphorus (TPP) enhance SOC (e.g., S_1_–T_1_ SOC coefficient: 3.30 cm^−1^ for TPP vs 1.31 cm^−1^ for DBDBD alone), accelerating ISC and stabilizing the green‐emitting triplet state.^[^ ^34^, ^35^, ^36^ ^]^ In contrast, CA lacks heavy atoms, resulting in weaker SOC (SOC coefficients 0.15 cm^−1^) and a redshifted emission due to slower ISC and enhanced vibrational relaxation.^[^ ^34^, ^35^, ^36^ ^]^ What is more, we calculated the electron transfer between the molecules (DBDBD:2MoBPA, DBD:2MoBPA, TPA:2MoBPA, DBDBD:CA, DBD:CA and TPA:CA) (Figure S12, Supporting Information). As shown in Figure S12 (Supporting Information), similar to DBDBD:TPP, when the host is 2MoBPA or CA, and the guest is TPA, DBD, or DBDBD, DBDBD:2MoBPA or DBDBD:CA still exhibits the highest number of transferred electrons (DBDBD:2MoBPA 0.104 eV, DBDBD:CA 0.044 eV). This further demonstrates the superior performance of the newly designed molecule DBDBD. Intriguingly, when TPP served as the host, electrons were transferred from the host to the guest (TPP→guest), whereas the reverse direction (guest→host) was observed for hosts TPA, 2MoBPA, and CA. This phenomenon may be attributed to the critical role of P atoms in modulating electron distribution.

**Figure 4:**
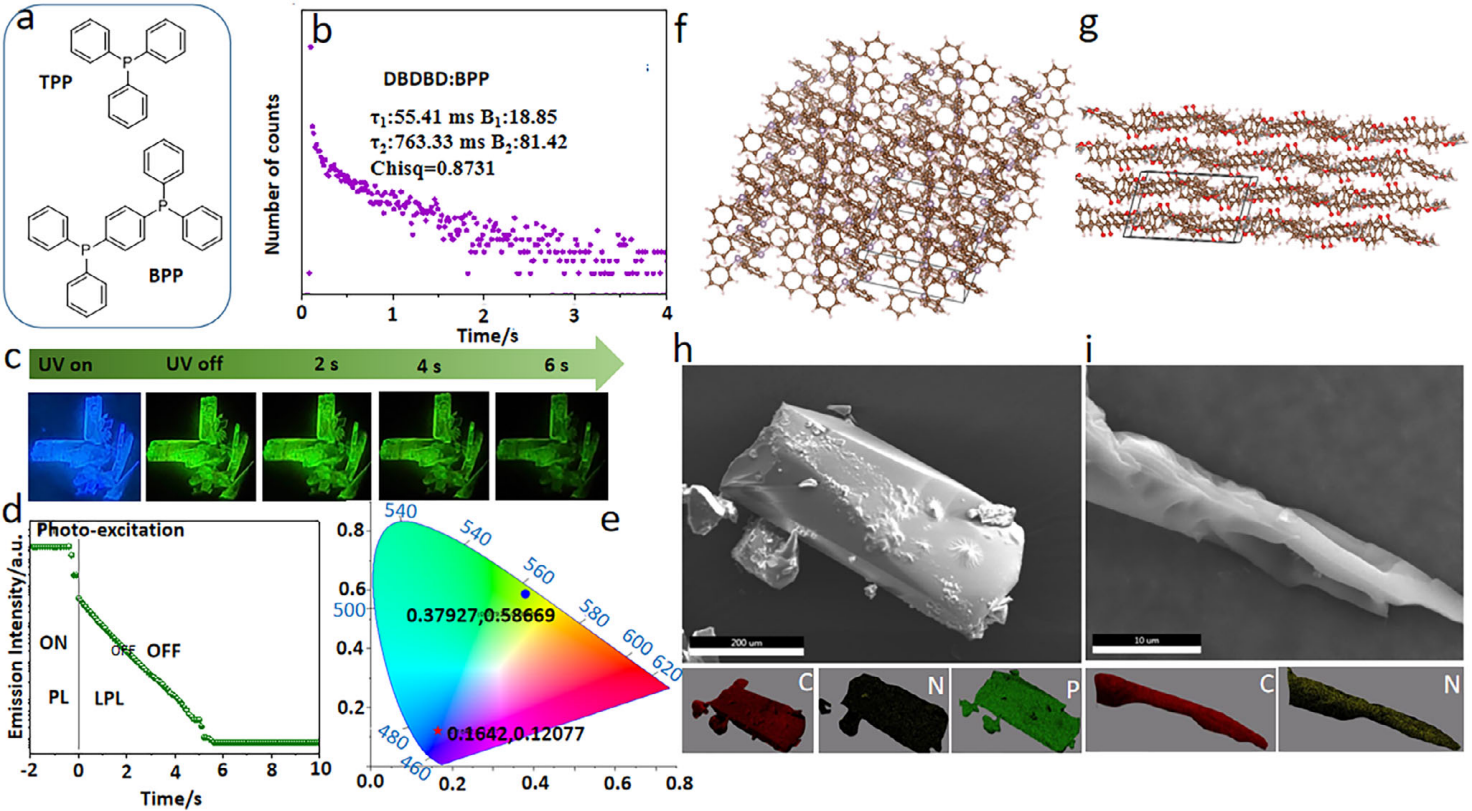
a) Chemical structures of TPP and BPP. b) Phosphorescence emission decay spectra for the doped crystals DBDBD:BPP (excitation: 365 nm). c) the LPL photographs of the doped crystal (DBDBD: BPP) under ambient conditions (excitation: 365 nm). d) Semi‐logarithmic plot of the emission decay profiles for the two doped crystals, showing photoluminescence (PL) from −2 to 0 s and long‐persistent luminescence (LPL) from 0 to 10 s after excitation is turned off (excitation wavelength: 365 nm; excitation power: 10 mW; excitation time: 2 s; and sample temperature: 300 K). e) Emission colors in the CIE 1931 chromaticity diagram: PL (star) and LPL (triangle) for DBDBD: BPP. f,g) Partial view of a 3D packing structure of the DBDBD:BPP and DBDBD:TPA single crystals. h,i) SEM image and corresponding EDX mappings of the DBDBD:BPP and DBDBD:TPA crystals.

Accordingly, we selected 1,4‐bis(diphenylphosphaneyl)benzene (BPP)‐a compound with a higher P‐atom content‐as the host to prepare the doped material DBDBD:BPP (**Figure** **4**a). As anticipated, DBDBD:BPP exhibited enhanced green afterglow brightness, a prolonged afterglow time of 6 s (Figure 4c), and significantly improved room‐temperature phosphorescence lifetime (763.33 ms) (Figure 4b) and green colour (Figure 4c), the ultraviolet, fluorescence and phosphorescence spectra of DBDBD:BPP are similar to those of DBDBD:TPP (Figure S13, Supporting Information), unequivocally demonstrating the synergistic effect of host‐guest electronic interactions on afterglow performance.

### 2.5 Molecular Packing and Structure‐Property Relationship

Considering that crystallization properties may also be one of the factors affecting the persistence properties of different doped crystals. The X‐ray diffraction (XRD) spectra of DBDBD:BPP, DBDBD:TPA, DBDBD:2MoBPA, and DBDBD:CA were obtained experimentally (Figure S1e, Supporting Information). All materials exhibited sharp diffraction peaks and flat baselines, indicating good crystallinity and highly ordered arrangement. Introducing a small amount of the donor DBDBD molecules was favorable to the crystallized crystals of TPP, TPA, 2MoBPA and CA.

To further explore the afterglow properties of the doped crystals, we first analyzed their single‐crystal structure and crystal topography (Figure 4f,g; Tables S10 and S11, Supporting Information). The doped DBDBD:BPP and DBDBD:TPA with the best (DBDBD:BPP) and the worst (DBDBD:TPA) afterglow properties were elected for preparing the single crystals to characterize the influence of guest molecules on the stacking and arrangement of host molecules. The profound disparity in RTP performance between DBDBD:BPP and DBDBD:TPA is directly attributable to their distinct molecular packing architectures, as unequivocally determined by the single‐crystal X‐ray diffraction results.

The exceptional afterglow duration (6 s) and phosphorescence lifetime (*τ* = 763.33 ms) of DBDBD:BPP are a direct consequence of its centrosymmetric P2_1_/n packing and efficient molecular confinement (Figure 4f; Table S10, Supporting Information). The large molecular volume (Vm = 1159.48 Å^3^) of DBDBD:BPP signifies that the bulky BPP host can construct a rigid, protective scaffold. This architecture drastically immobilizes the DBDBD guest, thereby suppressing vibrational and rotational non‐radiative pathways. This immobilization is the primary contributor to the system's ultralong phosphorescence. Conversely, the negligible afterglow (1 s) and short phosphorescence lifetime (*τ* = 386.38 ms) of DBDBD:TPA are rooted in its non‐centrosymmetric Cc lattice (Figure 4f; Table S10, Supporting Information). Its significantly bigger molecular volume (Vm = 5294.8 Å^3^) reveals a loose packing mode that fails to provide adequate rigidification. The absence of a symmetry center further permits molecular arrangements that promote non‐radiative decay, resulting in rapid triplet state quenching. Therefore, the order‐of‐magnitude enhancement in RTP performance is directly attributable to the superior crystalline confinement engineered within the BPP host matrix. This structure‐property relationship definitively establishes that optimized molecular packing‐characterized by high symmetry, little host volume, and rigid guest isolation‐is critical for realizing ultralong organic phosphorescence at room temperatures. To further investigate the elemental distribution in the doped crystals, we performed surface morphology analysis using scanning electron microscopy (SEM) and energy‐dispersive X‐ray spectroscopy (EDX) mapping (Figure 4h,i). In addition to carbon and nitrogen, phosphorus atoms were detected, which originated from the TPP molecules. The carbon, nitrogen, and phosphorus elements were uniformly distributed throughout the crystal, this shows that host and guest doping is relatively uniform.

**Figure 5:**
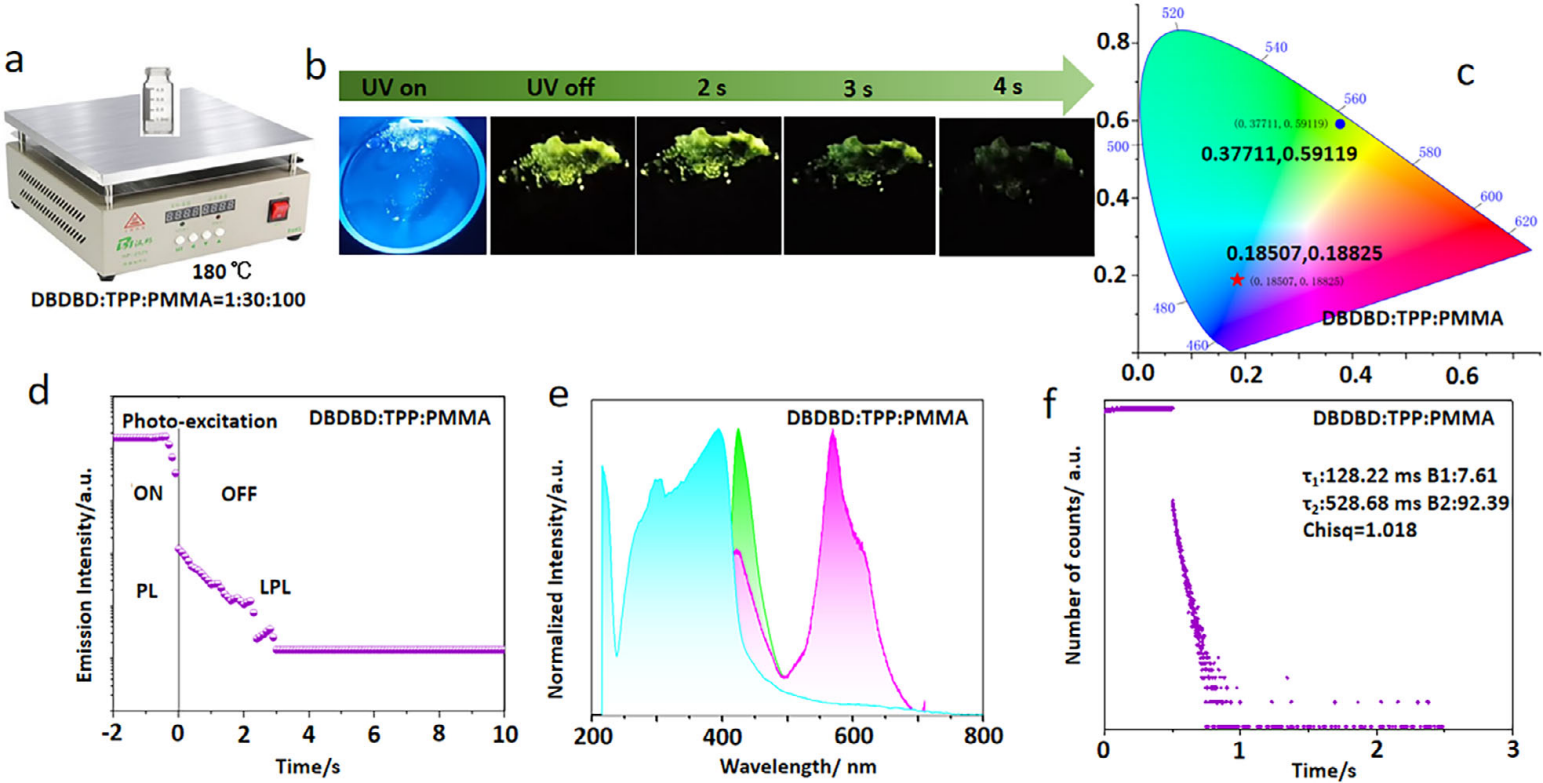
a) Preparation of films DBDBD: BPP:PMMA by heating method. b) the LPL photographs of the doped crystal (DBDBD:BPP:PMMA) under ambient conditions (excitation: 365 nm). c) Emission colors in the CIE 1931 chromaticity diagram: PL (star) and LPL (triangle) for DBDBD: BPP:PMMA. d) Semi‐logarithmic plot of the emission decay profiles for the two doped crystals, showing photoluminescence (PL) from −2 to 0 s and long‐persistent luminescence (LPL) from 0 to 10 s after excitation is turned off (excitation wavelength: 365 nm; excitation power: 10 mW; excitation time: 2 s; and sample temperature: 300 K). e) UV–vis absorption (blue) spectra, fluorescence (green), and phosphorescence (purple) emission spectra of the DBDBD: BPP:PMMA powder (excitation at λ_max_ of absorption), all were measured at room temperature. f) Phosphorescence emission decay spectra for the doped crystals DBDBD:BPP (excitation: 365 nm).

To better investigate the influence of a rigid environment on afterglow performance, we selected polymers as the substrate to construct a rigid environment and compared them with crystalline materials, as shown in **Figure** **5**. When we used traditional rigid material polymethyl methacrylate (PMMA) as the substrate, TPP as the host, and DBDBD as the guest material to construct the three‐component material DBDBD:TPP:PMMA, this material exhibited yellow‐green afterglow lasting ≈3.8 s (Figure 5a–c). The UV, fluorescence, and phosphorescence properties of DBDBD:TPP:PMMA were similar to those of DBDBD:TPP, but the phosphorescence lifetime and afterglow brightness of DBDBD:TPP:PMMA (528.68 ms, 92.39% and 128.22 ms, 7.61%) were lower than those of DBDBD:TPP (759.20 ms, 80.83% and 47.55 ms, 19.17%) (Figure 2e). This indicates that the crystalline material constructed via the simple solvent crystallization method exhibits superior afterglow performance, luminescence uniformity, and brightness compared to the rigid film formed through the more complex hot‐melt method, further demonstrating the advantages of crystalline afterglow materials.

### 2.6 Excellent Potential for Practical Applications Demonstrated

**Figure 6:**
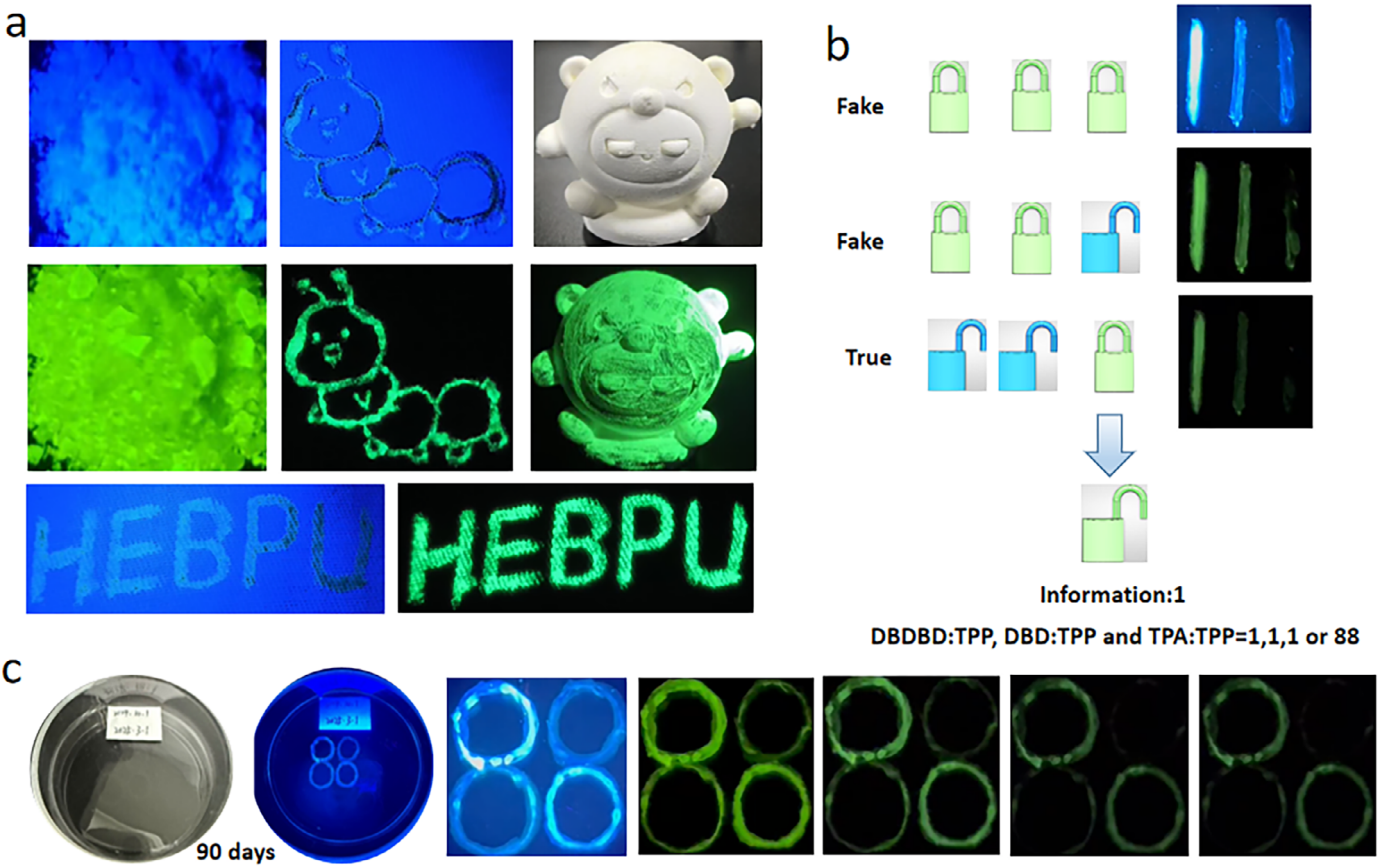
a) Photos of materials DBDBD: TPP crushed and coated on different substrates under ambient conditions, excited with 365 nm light at various temperatures. b) Illustration of multiple information encryption of “111” letters, based on the doped materials of TPA: TPP, DBD:TPP and DBDBD: TPP. c) Dynamic anti‐counterfeiting afterglow after 90 days of material placement (DBD:TPP and DBDBD: TPP).

Even more excitingly, when we ground the crystalline material, the afterglow effect remained excellent, as shown in **Figure** **6**a. This expands the potential applications of such materials. Figure 6 illustrates the applications of the time‐ and color‐tunable host‐guest doping system in fields such as anti‐counterfeiting displays and security protection. All films were produced using screen‐printing technology, with this doped material being printed onto various substrates. The material demonstrates excellent afterglow performance on diverse surfaces including paper, fabric, and plaster, indicating its broad applicability across different anti‐counterfeiting domains. Figure 6b demonstrates a multimodal data encryption strategy enabled by TPA:TPP, DBD:TPP and DBDBD:TPP modulation. The digits “111” were written on a quartz plate using TPA:TPP, DBD:TPP and DBDBD:TPP crystals. Leveraging the RTP properties responsiveness of these materials, stepwise information display and encryption were achieved. At UV on, all crystals emit visible light, revealing the complete “111” pattern. Upon removing the excitation source, the TPA:TPP crystal rapidly fades due to its lack of RTP, leaving only “11.” Over time, the emission from DBD:TPP also diminishes, and only DBDBD:TPP remains luminescent, displaying “1.” This host‐guest‐driven approach to multilevel, responsive data encryption offers a novel direction for advanced information security applications. Notably, this material exhibits remarkable air stability, maintaining superior afterglow performance even after 90 days of exposure to ambient air (Figure 6c).

## 3 Conclusion

In summary, we have developed a simple yet effective host‐guest doping strategy to achieve high‐performance, color‐tunable organic RTP with ultralong afterglow. The key to our success lies in the molecular design of butterfly‐shaped triphenylamine‐based guests (TPA, DBD, and DBDBD), which have systematically enhanced electron‐donating capabilities. By embedding these guests into the rigid, crystalline host matrices (TPP and BPP), we successfully obtained doped materials with tunable emission colors (from green to yellow) and remarkably long phosphorescence durations of up to 6 s under ambient conditions. Our comprehensive photophysical studies and DFT calculations reveal that the enhanced RTP performance arises from the following synergistic mechanisms: 1) The strong electron‐donating capability of the guests elevates the HOMO energy level, reduces the singlet‐triplet energy gap (ΔE_S‐T_), and promotes ISC; 2) Heavy‐atom effects from the host matrix (e.g., phosphorus atoms) significantly enhance SOC, further accelerating the ISC process; 3) Efficient host‐to‐guest energy transfer and a rigid crystalline environment collectively suppress non‐radiative decay and stabilize the triplet excitons. This work provides a versatile, low‐cost, and scalable alternative to complex molecular synthesis for designing advanced RTP materials. We believe the established structure–property relationship offers a general guideline for future molecular design, paving the way for next‐generation organic phosphors in optoelectronics and security technologies. While this study demonstrates efficient RTP modulation through host‐guest doping, the current systems are limited to green‐yellow emission; future work should explore broader spectral tuning (e.g., blue/red) by designing guests with extended conjugation or heavy‐atom‐free hosts. Further research is needed to optimize the trade‐off between phosphorescence lifetime and quantum yield, particularly for aqueous or flexible matrix applications, to expand their utility in bioimaging and wearable devices.

## 4 Experimental Section

### Instruments and Reagents

TPA, DBD, TPP, CA, BPP, and 2MoBPA were purchased from Henan Alpha Biochemical Technology Co., Ltd. DBDBD was synthesized according to the procedure detailed in the Supporting Information.

PMMA (average molecular weight: 1 30 000) were purchased from Tianjin Heowns Biochemical Technology Co., Ltd.

### Measurement and Characterization

The room‐temperature photoluminescence (PL), phosphorescence, and LPL spectra of the crystals were recorded with an Ocean Optics fiber spectrophotometer. UV–vis spectroscopy was performed using a Thermo Spectronic Helios Gamma spectrometer. Quartz cells had a path length of 1 cm. Fluorescence (FL) spectroscopy was carried using a Varian CARY ECLIPSE fluorescence spectrometer. Time‐resolved FL and PL experiments were performed with a spectrophotometer (Gilden Photonics) using a pulsed source at 480 nm (BDS‐SM ps diode laser). The time‐resolved signals were recorded by a time‐correlated single photon counting detection technique. Phosphorescence quantum yields were measured using an integrating sphere system (Edinburgh FLS980) with 365 nm excitation, calibrated against standard references. Samples were prepared as crystalline powders under nitrogen atmosphere to avoid oxygen quenching effects. Temperature‐dependent phosphorescence spectra were recorded using a closed‐cycle helium cryostat (77–373 K) with 365 nm excitation (FLS1000). Samples were mounted in a vacuum chamber to avoid condensation and oxygen quenching.

[CCDC 2 482 343 (DBDBD:BPP crystal) and 2 482 985 (DBDBD:TPA crystal) contains the supplementary crystallographic data for this paper. These data can be obtained free of charge from The Cambridge Crystallographic Data Centre via www.ccdc.cam.ac.uk/data_request/cif.]

### Experimental Process—Preparation of Doped Crystal Materials (TPA:TPP, DBD:TPP, DBDBD:TPP, DBDBD:TPA, DBDBD:2MoBPA, DBDBD:CA, DBDBD:BPP)

The guest material TPA, DBD, DBDBD (1 mg), host material TPP, 2MoBPA, CA, TPP and BPP (100 mg), dichloromethane (≈0.5 mL), and ethyl alcohol (≈3 mL) were added to a 5 mL crystal culture bottle. The mixture was stirred with a magnetic stirrer for ≈3 min. Subsequently, the mixture was cooled slowly at room temperature to allow crystals to precipitate. Once the crystals precipitated, they were filtered and placed in an oven for drying, which yielded large light crystals.

### Experimental Process—Preparation of Trace‐Doped DBDBD: TPP: PMMA

DBDBD, TPP, and PMMA were measured at a mass ratio of 1:30:100 and put into a 10 mL vial. The vial was then placed on a constant temperature heating table, heated and melted at 180 °C for 20 min, and cooled for later use.

### Experimental Process—Preparation of the DBDBD: TPP:PVP Paint

1.0 g of the trace‐doped (5 mol‰) DBDBD:TPP powder and 3.0 g of PVP were added into dichloromethane (8.0 mL), then the mixture was stirred at room temperature to produce the paint in which the mass percentage of host‐guest doping material was 25 wt.% (without solvent).

## Conflict of Interest

The authors declare no competing financial interest.

## Supporting information



Supporting Information

## Data Availability

The data that support the findings of this study are available in the supplementary material of this article.
