# Supplementary material for: Butterfly‐Shaped Guest Molecules Enable Tunable Room‐Temperature Phosphorescence in Host‐Guest Doped Systems
Source: Adv Sci (Weinh). 2025 Oct 8;13(2):e07940. doi: 10.1002/advs.202507940 (PMC12786342; doi:10.1002/advs.202507940)
Supplement: Supplementary file 1 — Supporting Information [file ADVS-13-e07940-s001.doc]

Supporting Information

Butterfly-Shaped Guest Molecules Enable Tunable Room-Temperature Phosphorescence in Host-Guest Doped Systems

Zongyong Lou^1^, Wenhui Feng^1*^, Kaining Wang^2^, Kun Gong^2^, Guangyu Wen^3^, and Denghui Ji^4**^

^1^ Hebei Petroleum University of Technology, Cheng De City 067000, P. R. China;

^2^School of Chemical Engineering and Technology, Tianjin University, Tianjin 300354, P. R. China;

^3^College of Physics and Hebei Advanced Thin Film Laboratory, Hebei Normal University, Shijiazhuang City, 050024, P. R. China;

^4^Science College, Shijiazhuang University, Shijiazhuang City 050035, P. R. China.

^*^E-mail: wenhuicuihua@126.com ^**^E-mail: [jidenghui2007@163.com](mailto:jidenghui2007@163.com)

**Table of contents**

[1. Characterization data ..S2](#__RefHeading___Toc373498800)

[2. Supplementary tables and figures S4](#__RefHeading___Toc373498801)

[3. References ……….. .S](#__RefHeading___Toc373498801)24

1. **Characterization data**

**TC-SPC**. Sample excitation was done with picosecond diode lasers (Horiba Jobin Yvon Instruments) at 321 nm or 375 nm, and the time resolution was ~ 150 ps. The laser pulse energy was ca. 15 pJ and attenuated (often by more than an order of magnitude) to the desired count rate of ca. 1% or less of the excitation frequency. A cooled (ca. -40°C) Hamamatsu MCP- photomultiplier R3809U 51 was used for detection of single photons, and the signal passed through a discriminator (Ortec 9307) and into a TAC (Ortec 566, 100 ns range used). The electrical trigger signal from the laser was also passed through a discriminator (Tennelec TC454) and on to the TAC (Ortec 566). The TAC output was read by a DAQ-1 MCA computer card using 1024 channels and collected with Horiba Jobin Yvon Data Station 2.5. Measurements were made in reverse mode at 5 MHz and under magic angle polarization. A cut-off filter, GG400 (Excitation at 321 nm) or GG 515 (Excitation at 375 nm), was used to block stray excitation light. A dilute solution of Ludox was used to record the instrument response function without any filter for solution measurements. No monochromator was used, i.e. all wavelengths transmitted by the cut-off filter were collected.

# Computational Methods

Utilizing the density functional theory, the calculations were executed through the DMol3 program^1-3^. The exchange-correlation functional employed was the generalized gradient approximation developed by Perdew, Burke, and Ernzerhof ^4^. This computational method mirrors the methodology used in our previous investigation of the interaction between polyacrylamide and H_2_O^5^. The basis set selected was Double Numerical plus Polarization, with the basis file defaulting to version 4.4. DFT semi-core pseudo potentials were configured as DFT-based potentials, and spin polarization was set to unrestricted. The quality of the global orbital cutoff was refined to fine, with a value of 3.7 Å, and a Fermi smearing of 0.005 Ha was applied. Additionally, we imposed the following convergence criteria: an energy tolerance of 1.0×10^-5^ Ha/atom, a self-consistent field tolerance of 1.0×10^-6^ Ha/atom, a maximum force tolerance of 0.002 Ha/Å, and a maximum displacement tolerance of 0.005 Å. Structures of TPA, DBD, DBDBD, TPP, BPP, 2MoBPA, and CA were optimized using the PBE functional with DNP (Double Numerical plus Polarization) basis set. Excited States (S_1_, T_1_): Singlet (S_1_) and triplet (T_1_) geometries were optimized using TD-DFT at the same level of theory. Frequency analyses confirmed all structures were at minima (no imaginary frequencies). SOC Matrix Elements: Calculated between singlet (Sn) and triplet (Tm) states using the Breit-Pauli operator. Spin components (Mₛ = 0, ±1) were evaluated to account for all possible transitions.


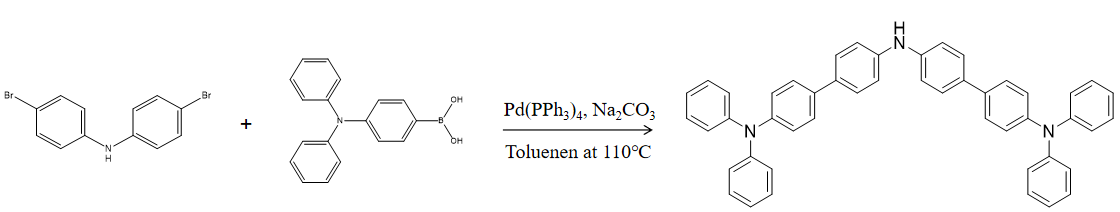


The synthetic route of the compound DBDBD

Synthesis of DBDBD. To a well degassed solution of 4-(diphenylamino)phenylboronic acid (0.54 g, 1.88 mmol) and bis(4- bromophenyl)amine (0.24 g, 0.75 mmol) and 2 M Na_2_CO_3_ (3 mL, 1.50 mmol) in toluene (10 mL) was added Pd(PPh_3_)_4_ (0.03 g, 0.03 mmol). The resulting mixture was stirred and heated to reflux at 110 °C for 48 h under argon atmosphere. After having been cooled to room temperature, the solvent was evaporated under reduced pressure and taken up with CH_2_Cl_2_. The organic layer was washed with brine and water sequentially and dried over anhydrous Na_2_SO_4_. After having been filtered, the solvent was evaporated to dryness and subjected to column chromatography on silica gel with petroleum/chloroform (2:1, v/v) as the eluent to give the product (0.42 g) in a yield of 80% as a white solid. ^1^ H NMR (400 MHz, CDCl_3_): δ 7.27 (dd, J=2.2, 1.7 Hz, 10H), 7.13−6.81 (d, J=6.8 Hz, 26H); ^13^C NMR (75 MHz, CDCl3): δ 153.04, 152.34, 138.29, 135.18, 133.01, 131.56, 129.05, 128.62, 127.15; MALDI-TOF: Calcd. for C_48_H_37_N_3_ 655.8; Found 655.5. High-Performance Liquid Chromatography (HPLC) analysis of DBDBD shows single peaks with no secondary phases (purity >99%) (Fig. S4).

1. **Supplementary table and figures**


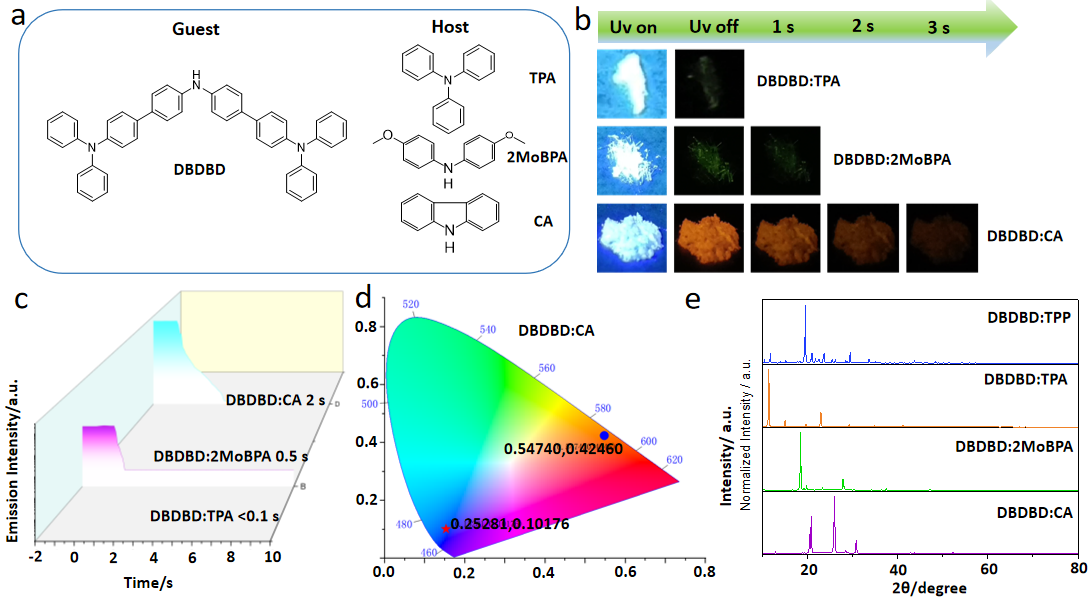


**Fig. S1** (a) Chemical structures of DBDBD, TPA, 2MoBPA and CA along with the LPL photographs of the doped crystal (DBDBD: TPA, DBDBD: 2MoBPA and DBDBD: CA) under ambient conditions (excitation: 365 nm)(b). (c) Semi-logarithmic plot of the emission decay profiles for the two doped crystals, showing photoluminescence (PL) from −2 to 0 s and long-persistent luminescence (LPL) from 0 to 10 s after excitation is turned off (excitation wavelength: 365 nm; excitation power: 10 mW; excitation time: 2 s; and sample temperature: 300 K). (d) Emission colors in the CIE 1931 chromaticity diagram: PL (star) and LPL (triangle) for DBDBD: CA. (e) XRD spectra of the doped crystals (a) DBDBD:TPP, (b) DBDBD : TPA, (c) DBDBD: 2MoBPA, and DBDBD: CA.


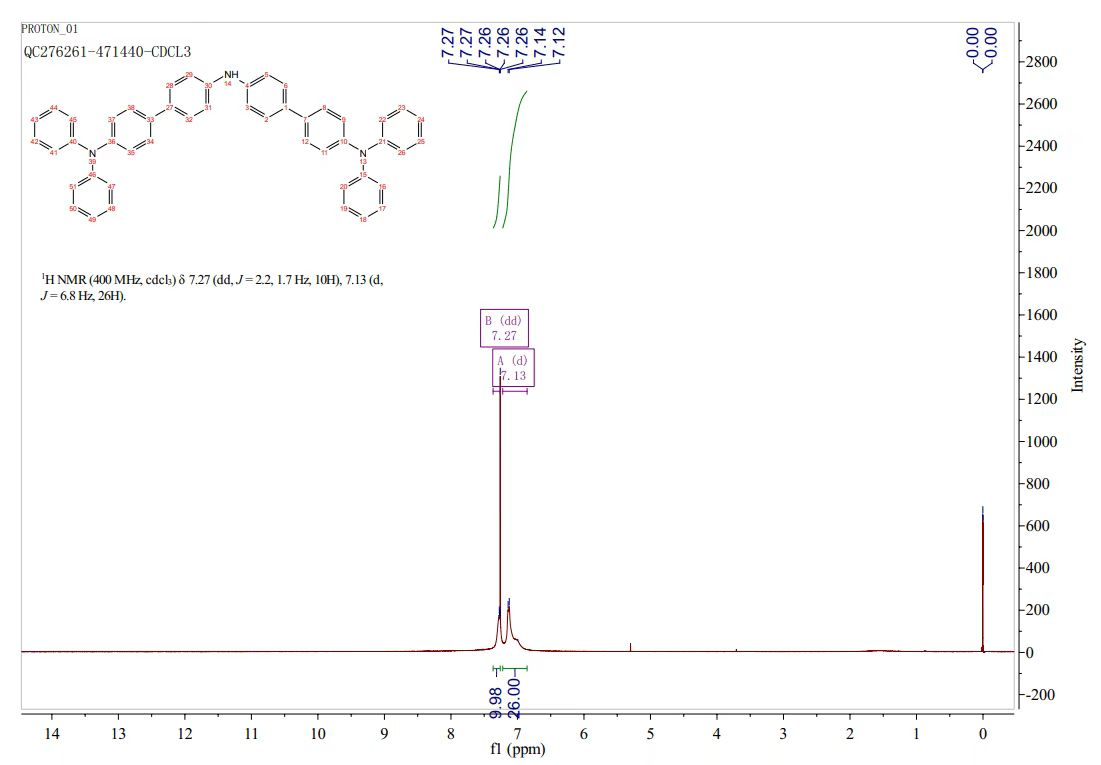


**Fig. S2** The nuclear magnetic spectrum of the compound DBDBD


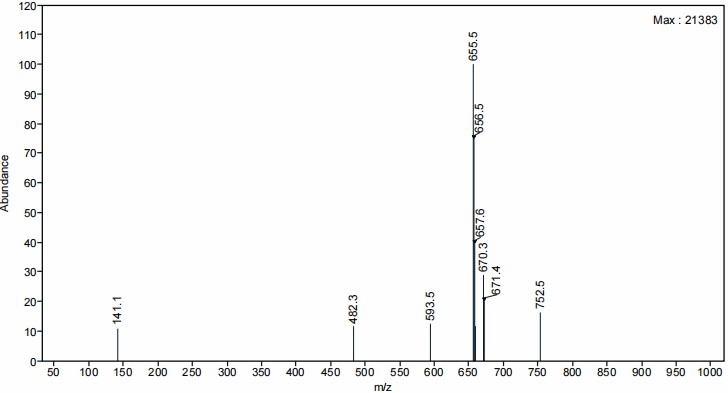


**Fig.S3** The high-resolution mass spectrometry of the compound DBDBD


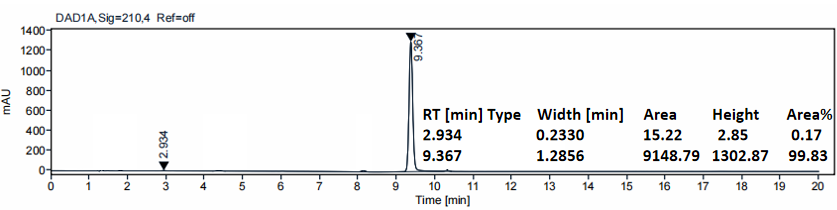


**Fig.S4** The High-Performance Liquid Chromatography of the compound DBDBD


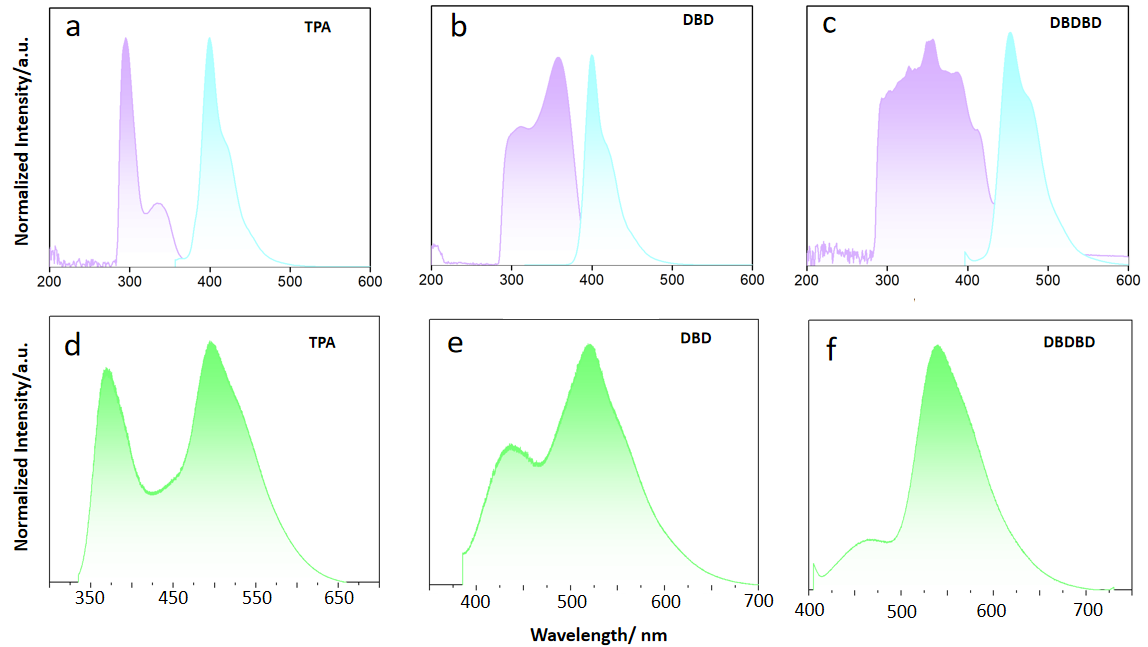


**Fig. S5** UV–Vis absorption (purple) and fluorescence (cyan) of the TPA (a), DBD (b) and DBDBD (c) measured at room temperature under ambient conditions. Phosphorescence (green) of the TPA (d), DBD (e) and DBDBD (f) measured at room temperature under ambient conditions.


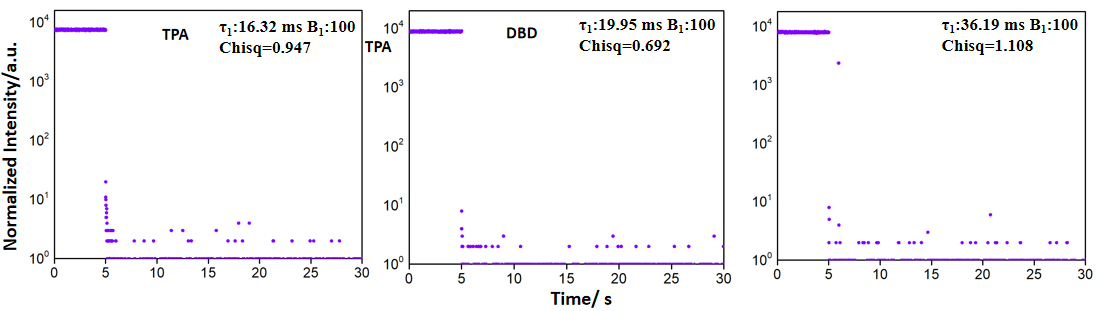


**Fig. S6**  Phosphorescence emission decay spectra of the TPA, DBD and DBDBD (Excitation: 365 nm).


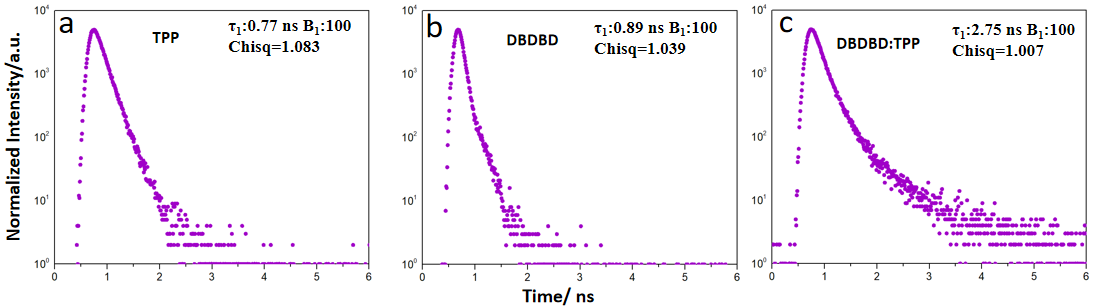


**Fig. S7**  Fluorescence emission decay spectra of the TPP, DBDBD and DBDBD:TPP (Excitation: 365 nm).


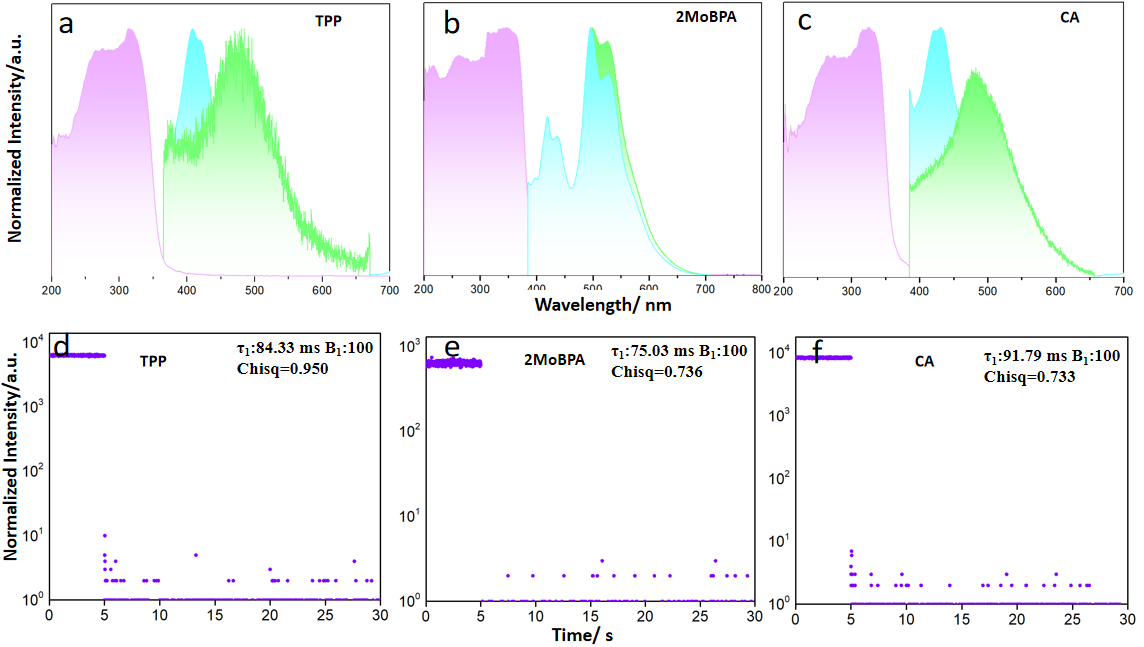


**Fig. S8** UV–Vis absorption (purple), fluorescence (cyan) and phosphorescence (green) of the TPP (a), 2MoBPA (b) and CA (c) measured at room temperature under ambient conditions. Phosphorescence emission decay spectra of the TPP (d), 2MoBPA (e) and CA (f) (Excitation: 365 nm).


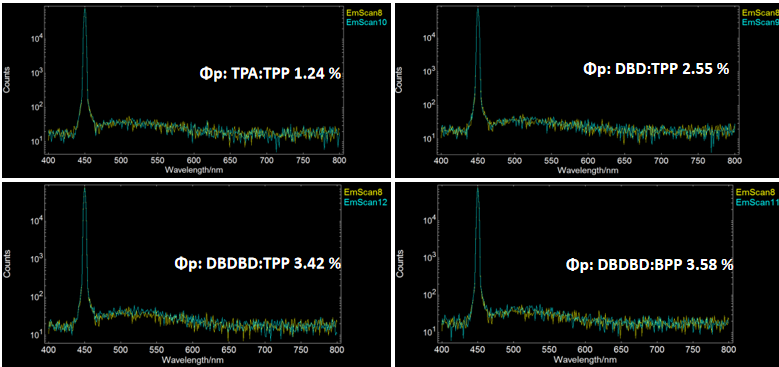


**Fig.S9** Phosphorescence quantum yield (Φₚ) of TPA:TPP, DBD:TPP, DBDBD:TPP, DBDBD:BPP at 298 K. Excitation at 450 nm. Data collected using an Edinburgh FLS980 spectrometer with a μs-delayed detection window.


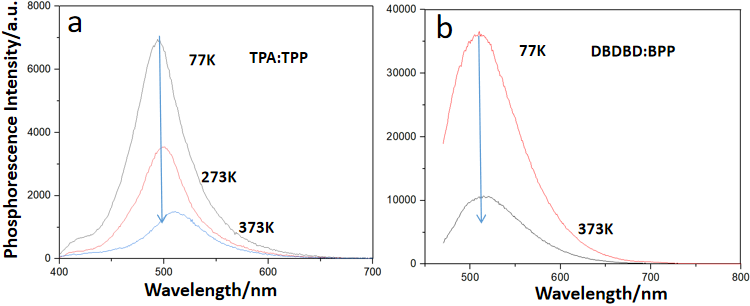


**Fig.S10** Temperature dependence of phosphorescence intensity for (a) TPA:TPP and (b) DBDBD:BPP. (Normalized phosphorescence spectra recorded from 77 K to 373 K under 365 nm excitation).


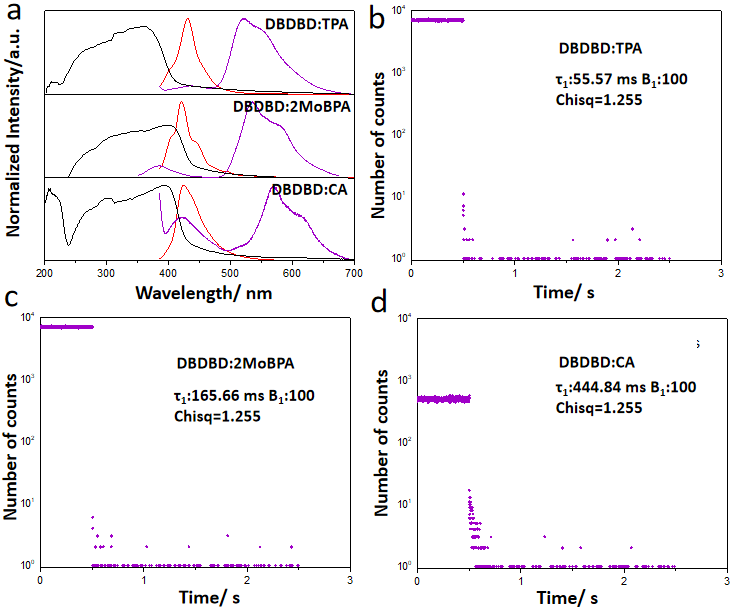


**Fig. S11** (a) UV-vis absorption (black) spectra, fluorescence (red), and phosphorescence (purple) emission spectra of the DBDBD:TPA, DBDBD:2MoBPA and DBDBD:CA powder (excitation at λmax of absorption), all were measured at room temperature. (b), (c) and (d) Phosphorescence emission decay spectra for the doped crystals DBDBD:TPA, DBDBD:2MoBPA and DBDBD:CA (excitation: 365 nm).


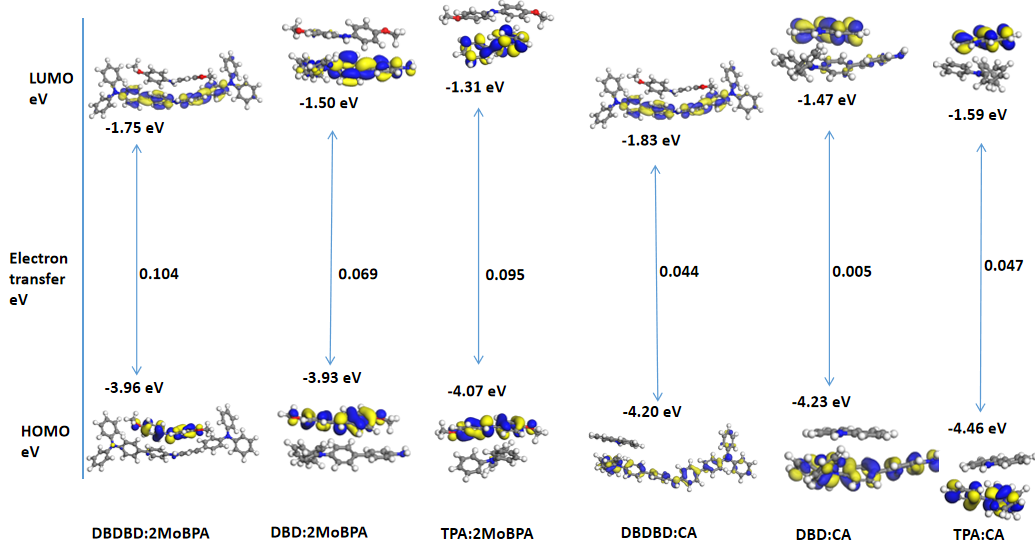


**Fig.S12** Electron transfer between host and guest and HOMO-LUMO of DBDBD:2MoBPA, DBD:2MoBPA, TPA:2MoBPA, DBDBD:CA, DBD:CA and TPA:CA using DMol3 program.


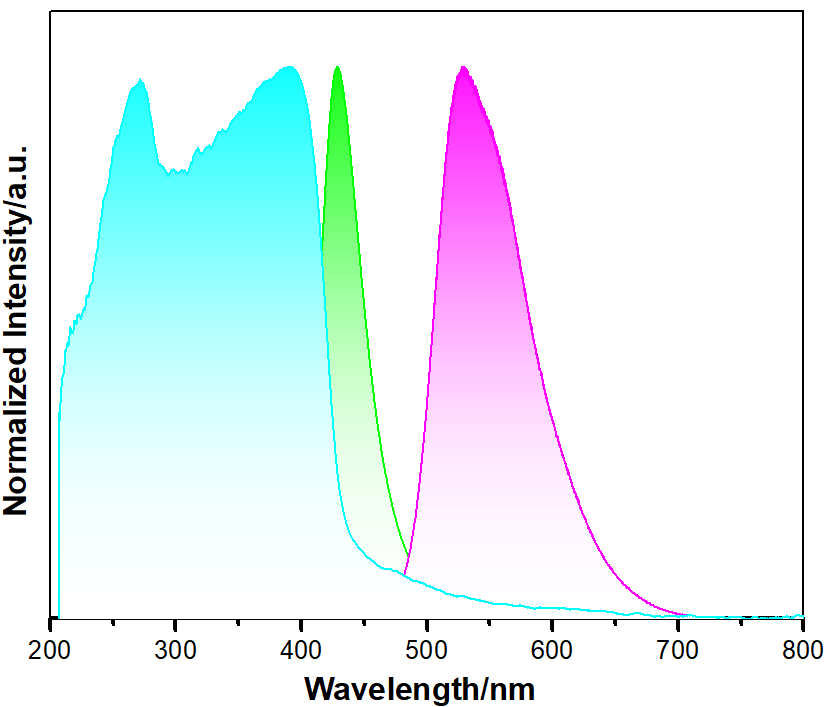


**Fig. S13** UV–Vis absorption (cyan), fluorescence (green) and phosphorescence spectrum (fuchsin) of the DBDBD:BPP measured at room temperature under ambient conditions.

Table S1 Some energy level data upon excitation for DBDBD and TPP.

| **sample** | **λ*_max_*(abs)**  **(nm)** | **λ*_max_*(fl)**  **(nm)** | **E(S_1_)^a)^**  **(eV)** | **λ*_max_*(ph)**  **(nm)** | **E(T_1_)^b)^**  **(eV)** |
| --- | --- | --- | --- | --- | --- |
| DBDBD | 375 | 460 | 2.97 | 546 | 2.28 |
| TPP | 330 | 405 | 3.37 | 490 | 2.53 |

1. Singlet energies E(S_1_) were calculated from absorption and fluorescence spectra of the crystals of T DBDBD and TPP, that is, E(S_1_) = 1241/[(λ_abs_+λ_fl_)/2].
2. Triplet energies E(T_1_) were due to phosphorescence spectra of the DDF-O, TPP2 and 2MoBPA crystals with the formula E(T_1_) = 1241/λ_ph_^[6]^.

Table S2 Excitation Energy: Energies (eV) of states Sₙ and Tₘ (e.g., S₁, T₂)

| **Energies (eV)** | **TPA** | **DBD** | **DBDBD** | **TPP** | **BPP** | **2MoBPA** | **CA** |
| --- | --- | --- | --- | --- | --- | --- | --- |
| S1 | 3.721 | 3.831 | 3.377 | 4.225 | 4.105 | 3.920 | 4.255 |
| S2 | 3.747 | 3.901 | 3.712 | 4.436 | 4.377 | 4.116 | 4.546 |
| S3 | 3.983 | 4.092 | 3.712 | 4.576 | 4.396 | 4.353 | 5.289 |
| S4 | 4.291 | 4.522 | 3.717 | 5.022 | 4.439 | 5.287 | 5.404 |
| S5 | 4.417 | 4.523 | 3.975 | 5.023 | 4.447 | 5.331 | 5.495 |
| T1 | 2.754 | 3.066 | 2.784 | 3.591 | 3.503 | 3.069 | 3.246 |
| T2 | 3.037 | 3.066 | 2.86 | 3.691 | 3.59 | 3.229 | 3.312 |
| T3 | 3.336 | 3.431 | 3.047 | 3.872 | 3.695 | 3.297 | 3.973 |
| T4 | 3.342 | 3.968 | 3.047 | 4.187 | 3.844 | 3.587 | 4.099 |
| T5 | 3.620 | 3.968 | 3.149 | 4.189 | 3.957 | 4.320 | 4.480 |

Table S3 Spin-Orbit Coupling (SOC) Matrix Elements & Coefficients of TPA

| **TPA** | **SOC Matrix Elements (cm⁻¹)** | | | | | | |
| --- | --- | --- | --- | --- | --- | --- | --- |
| **Transition (Sₙ-Tₘ)** | **Split by spin components Ms=0, ±1, Real (Re) and imaginary (Im) parts for each Mₛcomponent.** | | | | | | |
|  | Ms=0 | | Ms=-1 | | Ms=+1 | | SOC Coeff. |
|  | Re | Im | Re | Im | Re | Im |
| S0-T1 | 0.00 | -0.17 | -0.03 | 0.66 | -0.03 | -0.66 | 0.95 |
| S1-T1 | 0.00 | -0.18 | -0.58 | 0.00 | -0.58 | 0.00 | 0.84 |
| S2-T1 | 0.00 | 0.03 | 0.01 | -0.24 | 0.01 | 0.24 | 0.34 |
| S3-T1 | 0.00 | 1.27 | 0.22 | 0.00 | 0.22 | 0.00 | 1.31 |
| S4-T1 | 0.00 | -0.14 | 0.17 | -0.05 | 0.17 | 0.05 | 0.29 |
| S5-T1 | 0.00 | 0.01 | -0.07 | -0.01 | -0.07 | 0.01 | 0.10 |
| S0-T2 | 0.00 | 0.00 | -0.80 | 0.00 | -0.80 | 0.00 | 1.13 |
| S1-T2 | 0.00 | 0.01 | 0.00 | -0.71 | 0.00 | 0.71 | 1.00 |
| S2-T2 | 0.00 | 1.21 | -0.14 | 0.00 | -0.14 | 0.00 | 1.23 |
| S3-T2 | 0.00 | 0.00 | 0.00 | -0.32 | 0.00 | 0.32 | 0.45 |
| S4-T2 | 0.00 | 0.00 | 0.00 | -0.01 | 0.00 | 0.01 | 0.01 |
| S5-T2 | 0.00 | 0.08 | 0.02 | 0.13 | 0.02 | -0.13 | 0.20 |
| S0-T3 | 0.00 | -1.64 | -0.33 | 0.02 | -0.33 | -0.02 | 1.71 |
| S1-T3 | 0.00 | 0.00 | 0.01 | -0.02 | 0.01 | 0.02 | 0.03 |
| S2-T3 | 0.00 | 0.13 | 0.68 | 0.00 | 0.68 | 0.00 | 0.97 |
| S3-T3 | 0.00 | -0.01 | 0.00 | -0.78 | 0.00 | 0.78 | 1.10 |
| S4-T3 | 0.00 | 0.01 | 0.00 | -0.28 | 0.00 | 0.28 | 0.40 |
| S5-T3 | 0.00 | -0.01 | 0.01 | -0.23 | 0.01 | 0.23 | 0.33 |
| S0-T4 | 0.00 | -0.28 | -0.04 | -0.49 | -0.04 | 0.49 | 0.75 |
| S1-T4 | 0.00 | -0.27 | 0.31 | 0.00 | 0.31 | 0.00 | 0.51 |
| S2-T4 | 0.00 | 0.01 | -0.01 | 0.16 | -0.01 | -0.16 | 0.23 |
| S3-T4 | 0.00 | -0.84 | -0.15 | 0.01 | -0.15 | -0.01 | 0.87 |
| S4-T4 | 0.00 | 0.28 | -0.28 | -0.06 | -0.28 | 0.06 | 0.49 |
| S5-T4 | 0.00 | 0.09 | 0.29 | -0.04 | 0.29 | 0.04 | 0.42 |
| S0-T5 | 0.00 | -0.61 | 0.01 | -0.33 | 0.01 | 0.33 | 0.77 |
| S1-T5 | 0.00 | -0.02 | 0.00 | -0.13 | 0.00 | 0.13 | 0.18 |
| S2-T5 | 0.00 | -0.26 | 0.11 | -0.01 | 0.11 | 0.01 | 0.30 |
| S3-T5 | 0.00 | 0.00 | 0.00 | -0.05 | 0.00 | 0.05 | 0.07 |
| S4-T5 | 0.00 | -0.03 | -0.01 | -0.04 | -0.01 | 0.04 | 0.07 |
| S5-T5 | 0.00 | -0.01 | 0.01 | 0.23 | 0.01 | -0.23 | 0.33 |

Table S4 Spin-Orbit Coupling (SOC) Matrix Elements & Coefficients of DBD

| **DBD** | **SOC Matrix Elements (cm⁻¹)** | | | | | | |
| --- | --- | --- | --- | --- | --- | --- | --- |
| **Transition (Sₙ-Tₘ)** | **Split by spin components Ms=0, ±1, Real (Re) and imaginary (Im) parts for each Mₛcomponent.** | | | | | | |
|  | Ms=0 | | Ms=-1 | | Ms=+1 | | SOC Coeff. |
|  | Re | Im | Re | Im | Re | Im |
| S0-T1 | 0.00 | 0.00 | -0.62 | -0.50 | -0.62 | 0.50 | 1.13 |
| S1-T1 | 0.00 | 0.00 | -0.46 | 0.58 | -0.46 | -0.58 | 1.05 |
| S2-T1 | 0.00 | -0.34 | -0.22 | 0.18 | -0.22 | -0.18 | 0.53 |
| S3-T1 | 0.00 | 1.44 | 0.18 | 0.22 | 0.18 | -0.22 | 1.50 |
| S4-T1 | 0.00 | -0.26 | 0.16 | 0.08 | 0.16 | -0.08 | 0.36 |
| S5-T1 | 0.00 | -0.05 | -0.08 | 0.16 | -0.08 | -0.16 | 0.26 |
| S0-T2 | 0.00 | 0.00 | 0.49 | -0.61 | 0.49 | 0.61 | 1.11 |
| S1-T2 | 0.00 | 0.00 | -0.58 | -0.46 | -0.58 | 0.46 | 1.05 |
| S2-T2 | 0.00 | -1.44 | 0.18 | 0.22 | 0.18 | -0.22 | 1.50 |
| S3-T2 | 0.00 | -0.34 | 0.22 | -0.18 | 0.22 | 0.18 | 0.53 |
| S4-T2 | 0.00 | -0.05 | 0.08 | -0.16 | 0.08 | 0.16 | 0.26 |
| S5-T2 | 0.00 | 0.26 | 0.15 | 0.08 | 0.15 | -0.08 | 0.35 |
| S0-T3 | 0.00 | 1.62 | 0.00 | 0.00 | 0.00 | 0.00 | 1.62 |
| S1-T3 | 0.00 | 0.00 | 0.00 | 0.00 | 0.00 | 0.00 | 0.00 |
| S2-T3 | 0.00 | 0.00 | -0.34 | 0.72 | -0.34 | -0.72 | 1.13 |
| S3-T3 | 0.00 | 0.00 | -0.72 | -0.35 | -0.72 | 0.35 | 1.13 |
| S4-T3 | 0.00 | 0.00 | -0.02 | -0.03 | -0.02 | 0.03 | 0.05 |
| S5-T3 | 0.00 | 0.00 | -0.03 | 0.02 | -0.03 | -0.02 | 0.05 |
| S0-T4 | 0.00 | 0.01 | 1.09 | 0.38 | 1.09 | -0.38 | 1.63 |
| S1-T4 | 0.00 | 0.00 | 0.15 | -0.42 | 0.15 | 0.42 | 0.63 |
| S2-T4 | 0.00 | -0.02 | 0.25 | -0.09 | 0.25 | 0.09 | 0.38 |
| S3-T4 | 0.00 | -0.16 | -0.09 | -0.25 | -0.09 | 0.25 | 0.41 |
| S4-T4 | 0.00 | -0.19 | -0.10 | -0.11 | -0.10 | 0.11 | 0.28 |
| S5-T4 | 0.00 | -0.11 | 0.11 | -0.10 | 0.11 | 0.10 | 0.24 |
| S0-T5 | 0.00 | 0.00 | -0.38 | 1.09 | -0.38 | -1.09 | 1.63 |
| S1-T5 | 0.00 | 0.00 | 0.42 | 0.15 | 0.42 | -0.15 | 0.63 |
| S2-T5 | 0.00 | 0.16 | -0.09 | -0.25 | -0.09 | 0.25 | 0.41 |
| S3-T5 | 0.00 | -0.02 | -0.25 | 0.09 | -0.25 | -0.09 | 0.38 |
| S4-T5 | 0.00 | -0.11 | -0.11 | 0.10 | -0.11 | -0.10 | 0.24 |
| S5-T5 | 0.00 | 0.19 | -0.10 | -0.11 | -0.10 | 0.11 | 0.28 |

Table S5 Spin-Orbit Coupling (SOC) Matrix Elements & Coefficients of DBDBD

| **DBDBD** | **SOC Matrix Elements (cm⁻¹)** | | | | | | |
| --- | --- | --- | --- | --- | --- | --- | --- |
| **Transition (Sₙ-Tₘ)** | **Split by spin components Ms=0, ±1, Real (Re) and imaginary (Im) parts for each Mₛcomponent.** | | | | | | |
|  | Ms=0 | | Ms=-1 | | Ms=+1 | | SOC Coeff. |
|  | Re | Im | Re | Im | Re | Im |
| S0-T1 | 0.00 | -0.21 | -0.41 | 0.95 | -0.41 | -0.95 | 1.48 |
| S1-T1 | 0.00 | 0.35 | -0.66 | 0.60 | -0.66 | 0.60 | 1.31 |
| S2-T1 | 0.00 | 0.17 | -0.01 | 0.00 | -0.01 | 0.00 | 0.17 |
| S3-T1 | 0.00 | 0.00 | 0.53 | -0.03 | 0.53 | 0.03 | 0.75 |
| S4-T1 | 0.00 | 0.00 | 0.18 | 0.27 | 0.18 | -0.27 | 0.46 |
| S5-T1 | 0.00 | 0.00 | -0.32 | -0.41 | -0.32 | 0.41 | 0.74 |
| S0-T2 | 0.00 | -0.60 | 0.00 | 0.00 | 0.00 | 0.00 | 0.60 |
| S1-T2 | 0.00 | 0.00 | 0.03 | 0.08 | 0.03 | -0.08 | 0.12 |
| S2-T2 | 0.00 | 0.00 | 0.66 | 0.05 | 0.66 | -0.05 | 0.94 |
| S3-T2 | 0.00 | 0.12 | 0.01 | 0.00 | 0.01 | 0.00 | 0.12 |
| S4-T2 | 0.00 | -0.13 | 0.00 | 0.00 | 0.00 | 0.00 | 0.13 |
| S5-T2 | 0.00 | -1.09 | 0.00 | 0.00 | 0.00 | 0.00 | 1.09 |
| S0-T3 | 0.00 | 0.01 | 1.11 | -0.09 | 1.11 | 0.09 | 1.57 |
| S1-T3 | 0.00 | 0.91 | 0.98 | 0.01 | 0.98 | 0.01 | 1.66 |
| S2-T3 | 0.00 | 0.34 | 0.00 | 0.04 | 0.00 | -0.04 | 0.34 |
| S3-T3 | 0.00 | 0.01 | -0.02 | -0.69 | -0.02 | 0.69 | 0.98 |
| S4-T3 | 0.00 | -0.05 | 0.05 | 0.09 | 0.05 | -0.09 | 0.15 |
| S5-T3 | 0.00 | -0.01 | -0.01 | -0.33 | -0.01 | 0.33 | 0.47 |
| S0-T4 | 0.00 | -0.27 | 0.05 | 0.00 | 0.05 | 0.00 | 0.28 |
| S1-T4 | 0.00 | 0.03 | 0.09 | 0.20 | 0.09 | -0.20 | 0.31 |
| S2-T4 | 0.00 | 0.01 | -0.01 | -0.63 | -0.01 | 0.63 | 0.89 |
| S3-T4 | 0.00 | 0.01 | 0.00 | -0.04 | 0.00 | 0.04 | 0.06 |
| S4-T4 | 0.00 | 1.13 | 0.00 | 0.00 | 0.00 | 0.00 | 1.13 |
| S5-T4 | 0.00 | 0.26 | 0.00 | -0.01 | 0.00 | 0.01 | 0.26 |
| S0-T5 | 0.00 | 0.00 | -0.01 | 0.54 | -0.01 | -0.54 | 0.76 |
| S1-T5 | 0.00 | -0.12 | 0.00 | 0.00 | 0.00 | 0.00 | 0.12 |
| S2-T5 | 0.00 | -0.18 | -0.01 | 0.00 | -0.01 | 0.00 | 0.18 |
| S3-T5 | 0.00 | 0.00 | 0.39 | -0.11 | 0.39 | 0.11 | 0.57 |
| S4-T5 | 0.00 | 0.00 | 0.14 | 0.19 | 0.14 | -0.19 | 0.33 |
| S5-T5 | 0.00 | 0.00 | -0.28 | -0.21 | -0.28 | 0.21 | 0.49 |

Table S6 Spin-Orbit Coupling (SOC) Matrix Elements & Coefficients of TPP

| **TPP** | **SOC Matrix Elements (cm⁻¹)** | | | | | | |
| --- | --- | --- | --- | --- | --- | --- | --- |
| **Transition (Sₙ-Tₘ)** | **Split by spin components Ms=0, ±1, Real (Re) and imaginary (Im) parts for each Mₛcomponent.** | | | | | | |
|  | Ms=0 | | Ms=-1 | | Ms=+1 | | SOC Coeff. |
|  | Re | Im | Re | Im | Re | Im |
| S0-T1 | 0.00 | 0.01 | 10.25 | 1.29 | 10.25 | -1.29 | 14.61 |
| S1-T1 | 0.00 | 2.99 | -0.43 | 0.88 | -0.43 | -0.88 | 3.30 |
| S2-T1 | 0.00 | 3.33 | 0.89 | 0.42 | 0.89 | -0.42 | 3.61 |
| S3-T1 | 0.00 | 0.01 | 0.88 | 0.02 | 0.88 | -0.02 | 1.24 |
| S4-T1 | 0.00 | -1.03 | 0.01 | 0.02 | 0.01 | -0.02 | 1.03 |
| S5-T1 | 0.00 | -0.24 | -0.01 | 0.01 | -0.01 | -0.01 | 0.24 |
| S0-T2 | 0.00 | 0.00 | 1.22 | -10.26 | 1.22 | 10.26 | 14.61 |
| S1-T2 | 0.00 | 3.32 | -0.88 | -0.42 | -0.88 | 0.42 | 3.59 |
| S2-T2 | 0.00 | -3.00 | -0.43 | 0.89 | -0.43 | -0.89 | 3.31 |
| S3-T2 | 0.00 | -0.01 | 0.01 | -0.87 | 0.01 | 0.87 | 1.23 |
| S4-T2 | 0.00 | 0.25 | -0.01 | 0.01 | -0.01 | -0.01 | 0.25 |
| S5-T2 | 0.00 | -1.03 | -0.01 | -0.01 | -0.01 | 0.01 | 1.03 |
| S0-T3 | 0.00 | -0.01 | 0.08 | 0.02 | 0.08 | -0.02 | 0.12 |
| S1-T3 | 0.00 | 0.02 | 0.94 | 1.06 | 0.94 | -1.06 | 2.00 |
| S2-T3 | 0.00 | 0.03 | -1.04 | 0.95 | -1.04 | -0.95 | 1.99 |
| S3-T3 | 0.00 | -0.02 | 0.00 | 0.00 | 0.00 | 0.00 | 0.02 |
| S4-T3 | 0.00 | -0.01 | -0.27 | -0.27 | -0.27 | 0.27 | 0.54 |
| S5-T3 | 0.00 | 0.00 | -0.27 | 0.27 | -0.27 | -0.27 | 0.54 |
| S0-T4 | 0.00 | -0.01 | -17.75 | -17.02 | -17.75 | 17.02 | 34.78 |
| S1-T4 | 0.00 | -0.71 | 0.26 | -1.05 | 0.26 | 1.05 | 1.69 |
| S2-T4 | 0.00 | -7.70 | -1.04 | -0.26 | -1.04 | 0.26 | 7.85 |
| S3-T4 | 0.00 | -0.03 | -0.27 | -0.35 | -0.27 | 0.35 | 0.63 |
| S4-T4 | 0.00 | 1.32 | -0.03 | -0.50 | -0.03 | 0.50 | 1.50 |
| S5-T4 | 0.00 | -0.62 | 0.50 | -0.04 | 0.50 | 0.04 | 0.94 |
| S0-T5 | 0.00 | -0.02 | -16.89 | 17.64 | -16.89 | -17.64 | 34.54 |
| S1-T5 | 0.00 | -7.66 | 1.04 | 0.26 | 1.04 | -0.26 | 7.81 |
| S2-T5 | 0.00 | 0.69 | 0.26 | -1.04 | 0.26 | 1.04 | 1.67 |
| S3-T5 | 0.00 | 0.01 | -0.34 | 0.25 | -0.34 | -0.25 | 0.60 |
| S4-T5 | 0.00 | 0.61 | 0.50 | -0.05 | 0.50 | 0.05 | 0.94 |
| S5-T5 | 0.00 | 1.32 | 0.03 | 0.50 | 0.03 | -0.50 | 1.50 |

Table S7 Spin-Orbit Coupling (SOC) Matrix Elements & Coefficients of BPP

| **BPP** | **SOC Matrix Elements (cm⁻¹)** | | | | | | |
| --- | --- | --- | --- | --- | --- | --- | --- |
| **Transition (Sₙ-Tₘ)** | **Split by spin components Ms=0, ±1, Real (Re) and imaginary (Im) parts for each Mₛcomponent.** | | | | | | |
|  | Ms=0 | | Ms=-1 | | Ms=+1 | | SOC Coeff. |
|  | Re | Im | Re | Im | Re | Im |
| S0-T1 | 0.00 | -3.71 | 0.00 | -15.29 | 0.00 | 15.29 | 21.94 |
| S1-T1 | 0.00 | -3.72 | -0.05 | 0.79 | -0.05 | 0.79 | 3.88 |
| S2-T1 | 0.00 | 0.00 | -2.30 | 0.00 | -2.30 | 0.00 | 3.25 |
| S3-T1 | 0.00 | -4.32 | 0.00 | -0.80 | 0.00 | 0.80 | 4.47 |
| S4-T1 | 0.00 | 0.00 | -0.36 | 0.00 | -0.36 | 0.00 | 0.51 |
| S5-T1 | 0.00 | 1.54 | 0.00 | -2.14 | 0.00 | 2.14 | 3.40 |
| S0-T2 | 0.00 | 12.15 | 0.00 | -1.35 | 0.00 | 1.35 | 12.30 |
| S1-T2 | 0.00 | 0.00 | 2.18 | 0.00 | 2.18 | 0.00 | 3.08 |
| S2-T2 | 0.00 | 0.00 | -0.42 | 0.00 | -0.42 | 0.00 | 0.59 |
| S3-T2 | 0.00 | 1.85 | 0.00 | 0.54 | 0.00 | -0.54 | 2.00 |
| S4-T2 | 0.00 | 0.00 | -0.82 | 0.00 | -0.82 | 0.00 | 1.16 |
| S5-T2 | 0.00 | 1.20 | 0.00 | -0.58 | 0.00 | 0.58 | 1.45 |
| S0-T3 | 0.00 | 0.00 | 11.80 | 0.00 | 11.80 | 0.00 | 16.69 |
| S1-T3 | 0.00 | 2.72 | 0.00 | 0.06 | 0.00 | -0.06 | 2.72 |
| S2-T3 | 0.00 | 0.35 | 0.00 | 0.93 | 0.00 | -0.93 | 1.36 |
| S3-T3 | 0.00 | 0.00 | 1.40 | 0.00 | 1.40 | 0.00 | 1.98 |
| S4-T3 | 0.00 | 0.67 | 0.00 | -0.12 | 0.00 | 0.12 | 0.69 |
| S5-T3 | 0.00 | 0.00 | 2.25 | 0.00 | 2.25 | 0.00 | 3.18 |
| S0-T4 | 0.00 | 0.00 | 0.39 | 0.00 | 0.39 | 0.00 | 0.55 |
| S1-T4 | 0.00 | -0.16 | 0.00 | -0.80 | 0.00 | 0.80 | 1.14 |
| S2-T4 | 0.00 | 3.26 | 0.00 | -0.65 | 0.00 | 0.65 | 3.39 |
| S3-T4 | 0.00 | 0.00 | -0.28 | 0.00 | -0.28 | 0.00 | 0.40 |
| S4-T4 | 0.00 | 0.69 | 0.00 | 0.40 | 0.00 | -0.40 | 0.89 |
| S5-T4 | 0.00 | 0.00 | 0.07 | 0.00 | 0.07 | 0.00 | 0.10 |
| S0-T5 | 0.00 | -0.60 | 0.00 | -4.05 | 0.00 | 4.05 | 5.76 |
| S1-T5 | 0.00 | 0.00 | 0.30 | 0.00 | 0.30 | 0.00 | 0.42 |
| S2-T5 | 0.00 | 0.00 | 0.74 | 0.00 | 0.74 | 0.00 | 1.05 |
| S3-T5 | 0.00 | -1.50 | 0.00 | 0.96 | 0.00 | -0.96 | 2.02 |
| S4-T5 | 0.00 | 0.00 | 0.05 | 0.00 | 0.05 | 0.00 | 0.07 |
| S5-T5 | 0.00 | 1.63 | 0.00 | 0.50 | 0.00 | -0.50 | 1.78 |

Table S8 Spin-Orbit Coupling (SOC) Matrix Elements & Coefficients of 2MoBPA

| **2MoBPA** | **SOC Matrix Elements (cm⁻¹)** | | | | | | |
| --- | --- | --- | --- | --- | --- | --- | --- |
| **Transition (Sₙ-Tₘ)** | **Split by spin components Ms=0, ±1, Real (Re) and imaginary (Im) parts for each Mₛcomponent.** | | | | | | |
|  | Ms=0 | | Ms=-1 | | Ms=+1 | | SOC Coeff. |
|  | Re | Im | Re | Im | Re | Im |
| S0-T1 | 0.00 | -0.15 | 0.00 | -0.23 | 0.00 | 0.23 | 0.36 |
| S1-T1 | 0.00 | 0.00 | 0.41 | 0.00 | 0.41 | 0.00 | 0.58 |
| S2-T1 | 0.00 | -0.26 | 0.00 | -0.28 | 0.00 | 0.28 | 0.47 |
| S3-T1 | 0.00 | 0.00 | -0.34 | 0.00 | -0.34 | 0.00 | 0.48 |
| S4-T1 | 0.00 | 0.04 | 0.00 | 0.08 | 0.00 | -0.08 | 0.12 |
| S5-T1 | 0.00 | 0.00 | 0.89 | 0.00 | 0.89 | 0.00 | 1.26 |
| S0-T2 | 0.00 | 0.35 | 0.00 | 1.36 | 0.00 | -1.36 | 1.95 |
| S1-T2 | 0.00 | 0.00 | 0.24 | 0.00 | 0.24 | 0.00 | 0.34 |
| S2-T2 | 0.00 | 0.32 | 0.00 | -0.29 | 0.00 | 0.29 | 0.52 |
| S3-T2 | 0.00 | 0.00 | 0.19 | 0.00 | 0.19 | 0.00 | 0.27 |
| S4-T2 | 0.00 | 0.33 | 0.00 | 0.56 | 0.00 | -0.56 | 0.86 |
| S5-T2 | 0.00 | 0.00 | 0.99 | 0.00 | 0.99 | 0.00 | 1.40 |
| S0-T3 | 0.00 | 0.00 | 0.45 | 0.00 | 0.45 | 0.00 | 0.64 |
| S1-T3 | 0.00 | 0.13 | 0.00 | -0.90 | 0.00 | 0.90 | 1.28 |
| S2-T3 | 0.00 | 0.00 | 0.07 | 0.00 | 0.07 | 0.00 | 0.10 |
| S3-T3 | 0.00 | 0.13 | 0.00 | -0.18 | 0.00 | 0.18 | 0.29 |
| S4-T3 | 0.00 | 0.00 | -0.04 | 0.00 | -0.04 | 0.00 | 0.06 |
| S5-T3 | 0.00 | -0.43 | 0.00 | 0.29 | 0.00 | -0.29 | 0.59 |
| S0-T4 | 0.00 | 0.00 | -0.17 | 0.00 | -0.17 | 0.00 | 0.24 |
| S1-T4 | 0.00 | -0.48 | 0.00 | -0.61 | 0.00 | 0.61 | 0.99 |
| S2-T4 | 0.00 | 0.00 | 0.13 | 0.00 | 0.13 | 0.00 | 0.18 |
| S3-T4 | 0.00 | 0.38 | 0.00 | -0.51 | 0.00 | 0.51 | 0.82 |
| S4-T4 | 0.00 | 0.00 | -0.30 | 0.00 | -0.30 | 0.00 | 0.42 |
| S5-T4 | 0.00 | -0.94 | 0.00 | 0.92 | 0.00 | -0.92 | 1.61 |
| S0-T5 | 0.00 | 0.09 | 0.00 | -0.56 | 0.00 | 0.56 | 0.80 |
| S1-T5 | 0.00 | 0.00 | 0.05 | 0.00 | 0.05 | 0.00 | 0.07 |
| S2-T5 | 0.00 | 0.09 | 0.00 | 0.42 | 0.00 | -0.42 | 0.60 |
| S3-T5 | 0.00 | 0.00 | -0.07 | 0.00 | -0.07 | 0.00 | 0.10 |
| S4-T5 | 0.00 | 0.04 | 0.00 | -0.04 | 0.00 | 0.04 | 0.07 |
| S5-T5 | 0.00 | 0.00 | 0.41 | 0.00 | 0.41 | 0.00 | 0.58 |

Table S9 Spin-Orbit Coupling (SOC) Matrix Elements & Coefficients of CA

| **CA** | **SOC Matrix Elements (cm⁻¹)** | | | | | | |
| --- | --- | --- | --- | --- | --- | --- | --- |
| **Transition (Sₙ-Tₘ)** | **Split by spin components Ms=0, ±1, Real (Re) and imaginary (Im) parts for each Mₛcomponent.** | | | | | | |
|  | Ms=0 | | Ms=-1 | | Ms=+1 | | SOC Coeff. |
|  | Re | Im | Re | Im | Re | Im |
| S0-T1 | 0.00 | -0.04 | -0.11 | 0.05 | -0.11 | 0.05 | 0.18 |
| S1-T1 | 0.00 | 0.03 | -0.09 | 0.05 | -0.09 | 0.05 | 0.15 |
| S2-T1 | 0.00 | 0.04 | -0.07 | 0.03 | -0.07 | 0.03 | 0.11 |
| S3-T1 | 0.00 | 0.07 | -0.11 | 0.22 | -0.11 | 0.22 | 0.35 |
| S4-T1 | 0.00 | 0.05 | 0.13 | 0.21 | 0.13 | 0.21 | 0.35 |
| S5-T1 | 0.00 | -0.17 | -0.16 | 0.20 | -0.16 | 0.20 | 0.40 |
| S0-T2 | 0.00 | 0.11 | -0.03 | 0.02 | -0.03 | 0.02 | 0.12 |
| S1-T2 | 0.00 | 0.00 | -0.05 | 0.11 | -0.05 | 0.11 | 0.17 |
| S2-T2 | 0.00 | 0.02 | 0.00 | 0.00 | 0.00 | 0.00 | 0.02 |
| S3-T2 | 0.00 | 0.05 | 0.00 | 0.00 | 0.00 | 0.00 | 0.05 |
| S4-T2 | 0.00 | 0.00 | -0.05 | 0.00 | -0.05 | 0.00 | 0.07 |
| S5-T2 | 0.00 | -0.06 | 0.00 | 0.00 | 0.00 | 0.00 | 0.06 |
| S0-T3 | 0.00 | -0.16 | -0.08 | 0.18 | -0.08 | 0.18 | 0.32 |
| S1-T3 | 0.00 | 0.04 | 0.00 | 0.00 | 0.00 | 0.00 | 0.04 |
| S2-T3 | 0.00 | -0.01 | 0.00 | 0.00 | 0.00 | 0.00 | 0.01 |
| S3-T3 | 0.00 | 0.00 | -0.01 | 0.01 | -0.01 | 0.01 | 0.02 |
| S4-T3 | 0.00 | -0.03 | 0.00 | 0.00 | 0.00 | 0.00 | 0.03 |
| S5-T3 | 0.00 | 0.00 | 0.00 | 0.00 | 0.00 | 0.00 | 0.00 |
| S0-T4 | 0.00 | 0.00 | 0.00 | 0.00 | 0.00 | 0.00 | 0.00 |
| S1-T4 | 0.00 | -0.02 | -0.01 | 0.01 | -0.02 | 0.01 | 0.03 |
| S2-T4 | 0.00 | -0.01 | 0.00 | 0.00 | 0.00 | 0.00 | 0.01 |
| S3-T4 | 0.00 | -0.05 | 0.00 | 0.00 | 0.00 | 0.00 | 0.05 |
| S4-T4 | 0.00 | 0.00 | 0.00 | 0.00 | 0.00 | 0.00 | 0.00 |
| S5-T4 | 0.00 | 0.01 | 0.00 | 0.00 | 0.00 | 0.00 | 0.01 |
| S0-T5 | 0.00 | -0.07 | -0.17 | 0.05 | -0.17 | 0.15 | 0.30 |
| S1-T5 | 0.00 | -0.04 | -0.04 | 0.01 | -0.04 | 0.01 | 0.07 |
| S2-T5 | 0.00 | 0.06 | 0.00 | 0.00 | 0.00 | 0.00 | 0.06 |
| S3-T5 | 0.00 | 0.00 | 0.00 | 0.00 | 0.00 | 0.00 | 0.00 |
| S4-T5 | 0.00 | 0.00 | 0.00 | 0.00 | 0.00 | 0.00 | 0.00 |
| S5-T5 | 0.00 | -0.05 | 0.00 | 0.00 | 0.00 | 0.00 | 0.05 |

Table S10 Crystal data and structure refinement for DBDBD:BPP.

Identification code 2

Empirical formula C_30_ H_24_ P_2_

Formula weight 446.43

Temperature 99.99(10) K

Wavelength 1.54184 Å

Crystal system Monoclinic

Space group P _1_ _21_/n _1_

Unit cell dimensions a = 6.81153(19) Å a= 90°.

b = 11.9088(4) Å b= 97.070(3)°.

c = 14.4034(4) Å g = 90°.

Volume 1159.48(6) Å^3^

Z 2

Density (calculated) 1.279 Mg/m^3^

Absorption coefficient 1.806 mm^-1^

F(000) 468

Crystal size 0.24 x 0.22 x 0.14 mm^3^

Theta range for data collection 7.439 to 76.434°.

Index ranges -5<=h<=8, -14<=k<=12, -16<=l<=18

Reflections collected 4614

Independent reflections 2292 [R(int) = 0.0509]

Completeness to theta = 67.684° 98.7 %

Absorption correction Semi-empirical from equivalents

Max. and min. transmission 1.00000 and 0.61148

Refinement method Full-matrix least-squares on F^2^

Data / restraints / parameters 2292 / 0 / 145

Goodness-of-fit on F^2^ 1.096

Final R indices [I>2sigma(I)] R1 = 0.0526, wR2 = 0.1376

R indices (all data) R1 = 0.0663, wR2 = 0.1460

Extinction coefficient n/a

Largest diff. peak and hole 0.338 and -0.509 e.Å^-3^

Table S11 Crystal data and structure refinement for DBDBD:TPA.

Identification code 6

Empirical formula C_18_ H_15_ N

Formula weight 245.31

Temperature 100.00(10) K

Wavelength 0.71073

Crystal system Monoclinic

Space group C _1_ c _1_

Unit cell dimensions a = 15.4542(4) Å a= 90

b = 15.5875(4)Å b= 91.008(2)

c = 21.9832(5)Å g = 90

Volume 5294.8(2) Å^3^

Z 16

Density (calculated) 1.231 Mg/m^3^

Absorption coefficient 0.071 mm^-1^

F(000) 2080

Crystal size 0.22 x 0.22 x 0.22 mm^3^

Theta range for data collection 3.363 to 30.507°

Index ranges -20<=h<=21, -22<=k<=21, -29<=l<=31

Reflections collected 30774

Independent reflections 12202 [R(int) = 0.0327]

Completeness to theta = 25.242? 99.6 %

Absorption correction Semi-empirical from equivalents

Max. and min. transmission 1.00000 and 0.93588

Refinement method Full-matrix least-squares on F^2^

Data / restraints / parameters 12202 / 2 / 685

Goodness-of-fit on F2 1.051

Final R indices [I>2sigma(I)] R1 = 0.0356, wR2 = 0.0812

R indices (all data) R1 = 0.0420, wR2 = 0.0832

Absolute structure parameter -0.6(10)

Extinction coefficient n/a

Largest diff. peak and hole 0.183 and -0.203 e.Å^-3^

References

[1] B. Delley, J. Chem. Phys. 1990, 92, 508.

[2] B. Delley, J. Phys. Chem. 1996, 100, 6107.

[3] B. Delley, J. Chem. Phys. 2000, 113, 7756.

[4] Perdew J P, Burke K, Ernzerhof M. Physical review letters, 1996, 77(18): 3865.

[5] Wei T, Ren Y, Li Z. Chemical Engineering Journal, 2022, 434: 134646.

[6] Zhang Y D, Wu Y, Xu Y, Wang Q, Liu K, Chen J W, Cao J J, Zhang C, Fu H, H. L. Zhang, J.

Am. Chem. Soc. 2016, *138*, 6739-6745.
